# Next-Generation Cognitive-Behavioral Therapy for Depression: Integrating Digital Tools, Teletherapy, and Personalization for Enhanced Mental Health Outcomes

**DOI:** 10.3390/medicina61030431

**Published:** 2025-02-28

**Authors:** Evgenia Gkintoni, Stephanos P. Vassilopoulos, Georgios Nikolaou

**Affiliations:** Department of Educational Sciences and Social Work, University of Patras, 26504 Patras, Greece; stephanosv@upatras.gr (S.P.V.); gnikolaou@upatras.gr (G.N.)

**Keywords:** CBT, depression, digital tools, teletherapy, personalized approaches, Web-based interventions, treatment efficacy, AI-driven chatbots, clinical outcomes, mental health, psychotherapy, quality of life, user engagement

## Abstract

*Background and Objectives:* This systematic review aims to present the latest developments in next-generation CBT interventions of digital support tools, teletherapies, and personalized treatment modules in enhancing accessibility, improving treatment adherence, and optimizing therapeutic outcomes for depression. *Materials and Methods:* This review analyzed 81 PRISMA-guided studies on the efficacy, feasibility, and applicability of NG-CBT approaches. Other important innovations include web-based interventions, AI-operated chatbots, and teletherapy platforms, each of which serves as a critical challenge in delivering mental health care. Key messages have emerged regarding technological readiness, patient engagement, and the changing role of therapists within the digital context of care. *Results:* Findings indicate that NG-CBT interventions improve treatment accessibility and engagement while maintaining clinical effectiveness. Personalized digital tools enhance adherence, and teletherapy platforms provide scalable and cost-effective alternatives to traditional therapy. *Conclusions:* Such developments promise great avenues for decreasing the global burden of depression and enhancing the quality of life through novel, accessible, and high-quality therapeutic approaches.

## 1. Introduction

Cognitive-behavioral therapy (CBT) is a well-established and effective treatment for depression. However, traditional CBT faces limitations that may restrict its widespread dissemination and contribute to health disparities [1]. Next-generation CBT (NG-CBT) has emerged as a potential solution, incorporating synchronous teletherapy, web-based platforms, artificial intelligence (AI), and data analytics to deliver a more personalized and accessible approach to address the heterogeneity of depression [2,3]. This aligns with recent research exploring innovative ways to enhance CBT through digital tools and teletherapy, demonstrating the feasibility and efficacy of technology-enhanced interventions [4]. Web-based interventions are, without a doubt, the next step in the treatment of depression. NG-CBT, a new example of a web-based intervention, allows for large-scale and personalized depression care while alleviating significant economic burdens. In addition to web-based technology, NG-CBT integrates multimodal approaches, emphasizes the importance of the therapeutic alliance, and promotes adherence. However, while digital CBT expands accessibility, barriers related to digital literacy and privacy concerns must be addressed.

Digital literacy plays a crucial role in the successful implementation of NG-CBT. Many individuals, particularly older adults, those from lower socioeconomic backgrounds, and people with cognitive impairments, may struggle with the technological requirements of digital therapy platforms. Without adequate digital literacy skills, users may struggle to navigate online therapy modules, interact with AI-driven chatbots, and effectively engage in teletherapy sessions. To mitigate this issue, user-friendly platform designs, step-by-step onboarding guides, and digital literacy training should be considered part of NG-CBT interventions [5].

Privacy and data security concerns also pose significant challenges to the adoption of digital mental health interventions. As NG-CBT relies on teletherapy, mobile applications, and AI-driven tools, patients may be hesitant to disclose sensitive mental health information due to fears of data breaches, unauthorized access, or ethical concerns related to AI-based therapy. Ensuring end-to-end encryption, compliance with global data protection regulations (such as GDPR and HIPAA), and transparent privacy policies can help build user trust. Ethical considerations surrounding AI-driven decision-making in therapy should also be addressed to ensure patient confidentiality and ethical responsibility in digital mental health solutions [6,7].

Moreover, depression is the most significant contributor to worldwide disability and a leading cause of diminished quality of life and productivity [8], affecting over 280 million people across all age groups and socioeconomic backgrounds. The World Health Organization (WHO) estimates that depression accounts for a substantial portion of the global disease burden, with prevalence rates varying across regions due to sociocultural, economic, and healthcare disparities. However, the availability of evidence-based therapies is significantly lower than the demand for mental health care [9]. Digital tools may increase the financial benefits of psychological therapies, diminish the influence of geographic barriers, and improve treatment dissemination. Significantly, it may also complement face-to-face therapy [10]. Despite the extensive benefits of these digital tools, building a fully integrated treatment strategy has not been previously discussed, and combining these multiple approaches may standardize treatment methods that are typically not integrated [11,12,13].

Despite these challenges, digital tools refer to evidence-based Internet training programs, psychoeducation websites, commercial applications, or chatbots using conversational agents that are structured on CBT methods. Researchers did not specifically include a classification or detailed list of digital tools beyond those addressed in previous reviews [14,15]. The key reason was that digital tools rapidly became outdated as developers continually adopted AI and VR, cognitive and emotion recognition, voice analysis, and other related technologies to increase the accuracy of the services provided. In the case of teletherapy, interventions were designed to generalize traditional face-to-face CBT delivery approaches for depressive patients with videoconference systems and remarkably provide a patient-centered teletherapy protocol monitored by therapists throughout the blended treatment [16,17]. The integration of digital tools and the use of extended teletherapy will both emphasize personalized treatment strategies generated from the analysis of multiple patient social and biobehavioral markers [18,19].

This study aims to present a systematic review protocol to evaluate the technological aspects of NG-CBT, elucidate its conceptual framework, and address the primary research question regarding its feasibility and applicability in treating depression. The findings will inform researchers and clinicians about the potential of NG-CBT, highlight areas that need further development, and provide evidence to support future evidence-based depression care.

## 2. Literature Review

### 2.1. Understanding CBT

CBT is now the dominant therapy for depressive disorders and is also generally recommended in treatment guidelines for patients with major depression [20,21]. This is a compelling treatment for most people, and its efficacy extends to relapse prevention [22,23]. The key elements in traditional CBT for depression have to do with cognitive and behavioral activation. The focus lies on present and future cognitions, behaviors, and dysfunctional beliefs [24,25]. Through this process, patients identify and examine their beliefs and sentiments by testing evidence that confirms and disputes their dysphoric thoughts [26,27]. The consequences of these thoughts were documented in a daily thought log for further review.

Traditional CBT for depression has a few significant components. It begins by identifying problem areas through activity and mood monitoring, cognitive and behavioral event monitoring, and distorted thinking [28,29]. Next, patients work on specifying their behavioral goals, planning specific steps to achieve them, and receiving rewards based on completion. Other treatment components include social skill training, management of stressors, and assertion training. There is also a confrontation of interpersonal concerns [30,31]. The patient is supported interpersonally as the therapist encourages objective and assertive solutions to problems. These patients are then given assignments to chronicle their situations and thoughts about them, alone and with the therapist present. For instance, some have developed shortened forms; one of the shortened forms is approximately 10 min in length. These short forms have been shown to improve memory for treatment-related activities and have been effective even after the first therapeutic session [32,33,34,35].

CBT is a well-validated treatment for depression, and several randomized controlled trials (RCTs) have established its efficacy against depression. Meta-analyses have reported effect sizes for individually administered pure CBT ranging from medium to large for adults with mild to severe depression when delivered over an average of 11 to 20 sessions. Other meta-analyses have reported moderate to large effect sizes for individual and small-group delivery formats [36,37,38,39]. Evidence suggests that the most robust effects occur when CBT is delivered by a clinician experienced in the treatment. These meta-analyses have found that the benefits of pure CBT are like those offered by other psychotherapies with empirically validated efficacy in treating depression [40,41,42]. They have also found that CBT is only slightly less effective than the combined treatment of psychotherapy and pharmacotherapy [43,44,45]. However, the limitations of traditional face-to-face CBT are significant. These include the cost and inconvenience of treatment for patients and healthcare systems, the difficulty of finding trained providers, the underutilization of psychotherapy in general, and the quality of the treatment itself [46,47,48,49]. Moreover, patient preference is a critical factor in determining the best course of treatment for depression, and evidence suggests that a substantial proportion of patients prefer computerized interventions [50,51,52].

### 2.2. Digital Tools in Mental Health

In mental health, significant data-derived insights dynamically inform continuously adaptive support, while health promotion and prevention efforts reach people in all settings. Broader digital tools serve cognitive-emotional change, other psychological mechanisms, and delivery [53]. Daily assessment and reflection are available through short, flexible self-administration workflows, which, in addition to compliance, may enhance participants’ comprehension and motivation and potentially constitute pre-treatment cognitive-emotional reflection processes, improving therapeutic outcomes [54]. Indeed, when using machine learning models, we strongly encourage gender and ethno-racial diversity in the sample. Hence, the final services are adequate for users, different from the generally represented population [55]. Simple chatbots have made significant progress in delivering evidence-informed strategies addressing diverse issues such as mood, substance use, or social anxiety. Sophisticated versions are operational by providing empathetic engagement and understanding as they respond to language input, expressing states, thoughts, and behaviors, and guiding and encouraging users with varied skill levels [56].

Digital tools refer to Internet, mobile, or computer-based technologies that provide self-guided support programs or psychosocial treatment delivery channels. Various digital tools have been developed to facilitate access to mental health services by offering low-cost, convenient, and timely help from professionals or peers, as well as psychoeducational and therapeutic content. As a supplement to traditional mental health services, digital tools can provide extra strategies or monitoring opportunities for interveners to facilitate the implementation of their roles, which can help to decrease the increment of work. Such additional benefits make digital tools clinically significant in mental health promotion [57,58,59]. In addition, digital tools could increase the generalization of therapeutic techniques to clients’ daily living while decreasing clients’ dependency on therapists or waiting time for sessions. Therefore, it is not difficult to understand why applying digital tools to mental health programs is widespread [60,61]. According to their content features, various types of digital tools are available regarding mental health. For instance, psychoeducational websites provide mental health information, self-assessment tests, and self-help strategies to help users manage their mental health problems more efficiently [62,63]. Because of the simple program logic, psychoeducational websites require users’ autonomous reading and problem-solving skills to absorb the knowledge efficiently. Furthermore, computer-guided self-help interventions are developed as online eHealth or group-delivered interventions via guided mental health support groups. From moderator support models to postings in the discussion forum, users can access support anytime while participating in an eHealth program. Finally, different from computer-guided self-help interventions, peer social media platforms or suicide-related web forums use mutual aid and shared goal setting of support groups to enrich awareness, discussion, education, and referral services for users in need [64,65,66].

The primary benefits of integrating DCT are increasing access to CT for underserved patient populations, promoting adaptive learning and application of evidence-based techniques throughout the day, and enhancing clinical decision-making through passive data collection on patient outcomes and treatment processes [67,68]. Similarly, the advantages of combining DCT with tele-CT include overcoming transportation and scheduling barriers to receiving on-the-go support while increasing accountability and treatment compliance, building a strong therapeutic alliance, and enabling personalized therapist guidance remotely without technology-induced distance. Clinical applications of PF strategy can help CT cope with challenges due to symptom heterogeneity and discordance between core clinical strategies and patient needs [69,70]. However, the proposed NG-CT approach also must overcome substantial challenges related to technology readiness promotion, necessity and adequacy of risk assessment for DCT and PF, and therapist roles expanded by digitalized and personalized care. CT for depression is possible or even preferred in a digitalized format [71,72]. In contrast, DCT in either its discrete, EBT-incompatible stand-alone form or when complementing tele- or PST-based CT may be viewed as a groundbreaking step for increasing capacity for base-level care due to few qualified therapists for many patients, flexibility for several virtually demonstrated emotional diaries with real-life sensor integration, and readiness for online or blended treatment of patients need either proven EBT or more than technology-inducible skill training to engage in therapeutic activities [73,74,75,76,77].

### 2.3. Teletherapy in Mental Health

The Current State of Affairs Mental health care systems all over the world need to rethink many of their paradigms to face the increasing demand for adequate treatments for common mental disorders [78,79]. In this regard, telepsychology or teletherapy has emerged as a transformative means that could pave the way for the democratization of mental health care. Supporting the delivery of psychological care with the Internet and digital tools can help to transcend some of the traditional barriers that mental health care delivery faces [80,81]. Therapist training and availability, geographic constraints, and waiting time can all be alleviated by employing telepsychology in research and routine care. A vast amount of scientific evidence has already been published, showing that telepsychology is a valid and equivalent means for the delivery of mental health care for a variety of mental health problems [82,83]. These clinical interventions have comparable efficacy to face-to-face interventions. They can benefit specific populations, such as those looking to preserve anonymity or for flexibility in the time and availability of care [84,85].

The Use of Teletherapy in Traditional Psychological Interventions A meta-analysis of all CBT interventions delivered for depression through telepsychology and conducted mainly through synchronous group or individual formats, has shown that the asynchronous employment of digital tools supervised by clinicians was also effective in delivering CBT for depression, and the interventions were also effective in preventing depression symptoms. Moreover, the use of digital tools in CBT for depression has been frequently used in step-care interventions, showing that they are a valid means of delivering this type of intervention with positive results. It is also interesting to see how they were employed to maintain effective care at the end of the face-to-face wave of care, to ensure continuity of effective care delivery and availability in risky transition periods, or to deliver booster sessions to prevent relapse [86,87,88,89].

The Use of Teletherapy for Depression in Other New Psychological Interventions The results of studies that used telepsychology to deliver mindfulness-based interventions for depression generated mixed results, with some studies showing equivalence between them and other traditional face-to-face format, while other studies failed to show statistically significant effects in depressive symptoms or the specific mode of delivery-related outcomes [90,91,92]. Different models range from employing digital tools in all sessions to combined formats with face-to-face care to deliver these interventions. The use of a modular-like format in completed asynchronous support from a clinician was also shown to effectively prevent depression. Nonetheless, all these studies showed high dropout rates, possibly revealing how integrating different self-help components and pathways of care is needed to improve engagement and user retention in this mode of care delivery. The use of other forms of telepsychology, such as video teletherapy or therapeutic video games integrating CBT elements, is still scarce and is still a topic that needs significant investigation and integration in the era of P4 mental health care for depression [93,94,95].

### 2.4. Personalized Approaches in Mental Health

Personalized medicine is an approach to patient care that allows individual decisions regarding prevention, diagnosis, and therapy specific to the patient’s characteristics. The promise of personalized medicine includes more cost-effective care, improved patient outcomes, and fewer drugs and drug doses to invoke the fewest number and least severe side effects for optimal results. Interest in personalized medicine approaches for complex mental conditions, such as mood and anxiety disorders, is not new to the field of mental health [96,97,98]. However, it has experienced renewed interest given the recent discoveries that have identified different dimensions of psychopathology that may map onto more homogeneous populations than traditional symptom-based diagnostic criteria and/or be more tightly linked to differences in underlying etiological and pathophysiological processes than traditional diagnostic criteria [99,100,101].

A personalized approach to mental health means allowing everyone to find and connect with their most personal path to wellness. Mental health care has long since recognized that these paths, tailored to each person’s situation, preferences, and desired outcomes, will only marginally pass through clinical couches and care pathways. These highly personal paths will also require the exploitation of everyone’s social, family, and professional opportunities and challenges, as well as the communities in which they live. They often require the use of a wide range of personal resources, including the individual learning of new skills and coping strategies that seem to serve one person’s mental well-being better than those known by others. To the extent that a personalized pathway is personal, it will require much collaboration between the client and the supporter to build a trustful, effective helping relationship—whether between patient and caregiver, helper and help seeker, or peer to peer [102,103,104,105].

There is potential for personalization to add quality and efficiency to the treatment. For example, streamlining the intervention and avoiding the need for multiple cycles of depression screening and cognitive testing at the beginning, middle, and end of therapy [106,107,108,109,110] would be beneficial. By personalizing the treatment approach, more efficient and effective depression treatment may be made available to the population. The potential exists for a single treatment to adapt based on how the user is doing day-to-day, incorporate monitoring-based advice for which behavioral or cognitive technique to use next, and remind the user to perform key exercises to prompt the brain to naturally reassociate context, thus strengthening the downregulation of linked emotional networks [111,112,113]. This personalization would utilize digital tracking and app-based, on-demand guidance with minimal therapist touchpoints to support patient engagement and adherence [114,115,116].

This would be supported by therapist data dashboards that could provide insights to ensure that the treatment was effective and high quality for each patient, particularly for patients who, for some reason, deviate from the expected treatment path. For example, a woman with behavioral activation and cognitive restructuring sessions scheduled for one day may move through the former quickly while experiencing cognitive restructuring challenges, thereby preventing progress in the intervention [117,118,119]. In a digital navigation tool using minimal therapist touchpoints, associated pain or fear diagnostics and tracking can also achieve real-time monitoring. The ease and consistency of remote monitoring of pain and fear leave ample room to tweak the embedded perturbation rhythm, better personalizing the treatment for each person [120,121,122,123,124].

### 2.5. Research Questions

The rapid advancement of digital health technologies and the increasing need for accessible mental health care have led to the evolution of CBT (CBT) through the integration of digital tools, teletherapy, and personalized approaches. In addressing the rising prevalence of depression, next-generation CBT aims to leverage these innovations to improve treatment outcomes, enhance patient engagement, and overcome traditional barriers to mental health care. This section outlines the key research questions centrally to understanding the effectiveness and future potential of these innovations within the framework of CBT for depression. These questions are guided by existing research and aim to explore how technological advancements can be optimized to address individual patient needs, deliver scalable mental health solutions, and ensure both cost-effectiveness and efficacy in treating depression. The hypotheses and descriptions following each question further clarify the assumptions and goals underpinning this next-generation CBT approach.

[RQ1] How can technology, such as automated text messaging, enhance the delivery of CBT(CBT) for depression?

**Hypothesis 1.**  *It is hypothesized that technology, such as automated text messaging systems, will provide real-time, low-cost support that complements traditional CBT sessions by improving patient adherence and engagement. Studies suggest that patients using automated text messages as an adjunct to CBT may experience better treatment adherence and symptom monitoring compared to traditional CBT alone.*

[RQ2] What are the key challenges in integrating digital tools into traditional CBT, and how can they be addressed?

**Hypothesis 2.**  *Integrating digital tools into CBT may lead to accessibility issues, patient technology literacy, and privacy concerns. However, it is hypothesized that addressing these barriers through user-friendly designs and robust data protection strategies will enhance the effectiveness and acceptance of digital CBT platforms.*

[RQ3] What are the efficacy outcomes of teletherapy-based CBT compared to in-person therapy for depression?

**Hypothesis 3.**  *Teletherapy-based CBT is hypothesized to demonstrate similar, if not superior, efficacy outcomes to in-person therapy. Teletherapy provides flexibility, reduces barriers to access, and allows continuous support, which may enhance treatment outcomes, especially for patients in underserved areas.*

[RQ4] How can teletherapy delivery of CBT be optimized for personalized patient care?

**Hypothesis 4.**  *By incorporating patient-specific factors, such as the severity of depression, technological proficiency, and individual preferences, teletherapy CBT can be tailored to maximize efficacy. It is hypothesized that personalized teletherapy CBT will improve treatment satisfaction and outcomes compared to a one-size-fits-all teletherapy model.*

[RQ5] How can CBT be personalized using digital tools to improve both cost-effectiveness and accessibility for patients with depression?

**Hypothesis 5.**  *Personalized CBT interventions delivered via digital tools, such as computer-assisted therapy platforms, will reduce healthcare costs while improving accessibility. The hypothesis is that personalized digital CBT will allow for more targeted treatment, improve cost-effectiveness by reducing the number of required therapist hours, and enhance long-term patient outcomes.*

[RQ6] What role do mobile apps and digital platforms play in delivering next-generation CBT for depression?

**Hypothesis 6.**  *Mobile apps and digital platforms are expected to enhance the scalability and reach of CBT for depression. Compared to traditional CBT approaches, it is hypothesized that these tools will provide patients with real-time support, progress tracking, and interactive exercises that will improve treatment outcomes and patient engagement.*

[RQ7] How effective are current digital CBT tools in providing long-term treatment outcomes for depression?

**Hypothesis 7.**  *Digital CBT tools are expected to provide long-term treatment outcomes that are comparable to those of traditional face-to-face therapy. The hypothesis is that continuous digital engagement through tools like apps and platforms will support sustained symptom management and relapse prevention in patients with depression.*

These research questions and hypotheses aim to provide a structured approach for exploring the impact and efficacy of digital tools, teletherapy, and personalization in CBT for depression.

## 3. Materials and Methods

This systematic review aims to evaluate the integration of digital tools, teletherapy, and personalized approaches in next-generation CBT (NG-CBT) for depression. This study synthesizes evidence regarding the effectiveness, feasibility, and applicability of these approaches while adhering to PRISMA guidelines to ensure replicability and transparency. This study examines technological advancements, such as automated text messaging, e-CBT platforms, and teletherapy, address accessibility barriers and improve treatment outcomes. Furthermore, this review explores personalized therapeutic approaches, leveraging patient-specific data and machine learning models to enhance the tailoring of interventions to individual needs. By analyzing RCTs and other relevant studies, this research investigates the comparative efficacy of NG-CBT versus traditional CBT, the long-term outcomes of digital interventions, and the strategies required to overcome implementation challenges. This work provides a comprehensive synthesis of the evidence to inform the development of scalable and personalized mental health solutions for depression.

### 3.1. Analytical Search Process

**Figure 1:**
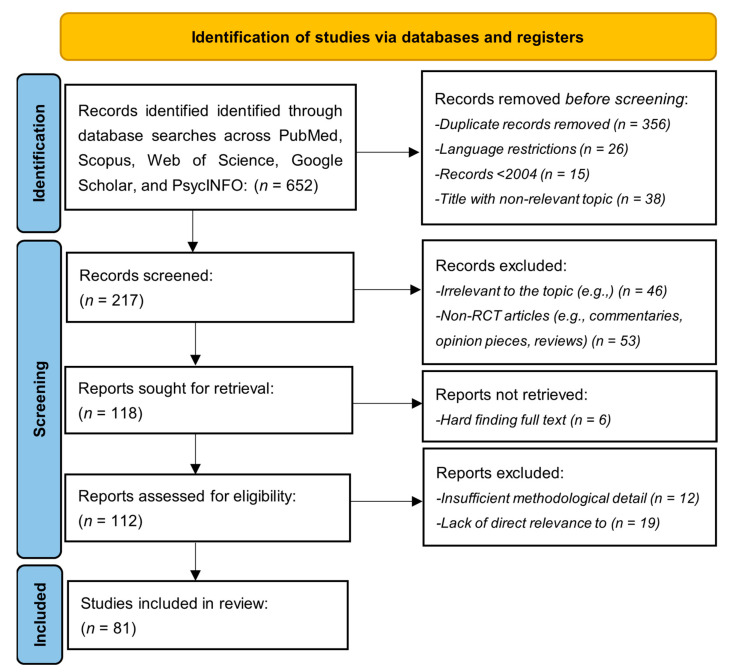
Flowchart of the PRISMA methodology.

This systematic review follows the PRISMA (Preferred Reporting Items for Systematic Reviews and Meta-Analyses) guidelines to ensure transparency and replicability in the synthesis of evidence [125]. A protocol outlining the objectives, eligibility criteria, information sources, and analysis methods was registered on Open Science Framework [https://osf.io/qap59, accessed on 25 February 2025|Registration DOI: 10.17605/OSF.IO/NSFP6] [126]. The analytical search began by identifying 652 records through database searches conducted across PubMed, Scopus, Web of Science, Google Scholar, and PsycINFO databases. Before the screening, 356 duplicate records were removed, along with 26 records due to language restrictions, 15 published before 2004, and 38 with non-relevant titles. After these removals, 217 records remained for further screening. During the screening phase, the titles and abstracts of 217 records were reviewed, and 46 were excluded as irrelevant to the topic. In contrast, 53 non-randomized controlled trial (RCT) articles, such as commentaries, opinion pieces, and reviews, were also excluded. This left 118 reports to be retrieved for further assessment. Of the 118 reports, six could not be retrieved due to difficulties in accessing the full text. The remaining 112 reports were assessed for eligibility, of which 12 were excluded for insufficient methodological detail and 19 for lacking direct relevance to the research question. Ultimately, 81 studies were included in this review (Table 1). This process reflects a systematic and rigorous approach to screening and selecting studies, ensuring transparency and adherence to the PRISMA guidelines. Eighty-one (n = 81) studies were included in the synthesis, providing the basis for further analysis (Figure 1).

### 3.2. Search Strategy

The search strategy for this systematic review was designed to comprehensively capture all relevant studies addressing NG-CBT for depression, integrating digital tools, teletherapy, and personalization. The search string was as follows:

(“Cognitive Behavioral Therapy” OR “CBT” OR “Next-Generation CBT” OR “NG-CBT”) AND (“Digital Tools” OR “Teletherapy” OR “Personalized Medicine”) AND (Depression OR Anxiety OR “Mental Health Outcomes”) AND (“Randomized Controlled Trial” OR “RCT”)

The search terms were customized for each database to ensure precise and comprehensive retrieval. Filters restricted the search to studies published in English from 2004 to 2024. Only RCTs investigating digital tools, teletherapy, or personalized approaches within CBT frameworks were included to ensure a focused selection of relevant studies.

### 3.3. Inclusion and Exclusion Criteria

The inclusion and exclusion criteria for this study were as follows:

Inclusion Criteria

- Studies investigating the efficacy, feasibility, or applicability of NG-CBT for depression.
- RCTs or other high-quality empirical studies.
- Studies employing digital tools, teletherapy, or personalized approaches in CBT interventions.
- Articles published in peer-reviewed journals after 2004 (to ensure relevance to recent advancements in digital mental health).
- Research written in English (to maintain consistency in the interpretation and methodological rigor).
- Full-text availability for a comprehensive review.

Exclusion Criteria

- Studies that did not focus on CBT or related interventions for depression.
- Non-empirical papers, such as reviews, commentaries, and opinion pieces.
- Articles published in languages other than English (to prevent interpretation inconsistencies and ensure the accessibility of findings).
- Research focused on populations or disorders outside the scope of depression treatment.
- Insufficient methodological detail or lack of direct relevance to NG-CBT.
- Studies published before 2004 are less applicable to current practice as significant advancements in digital CBT interventions have emerged in the past two decades.

### 3.4. Risk of Bias Assessment

**Figure 2:**
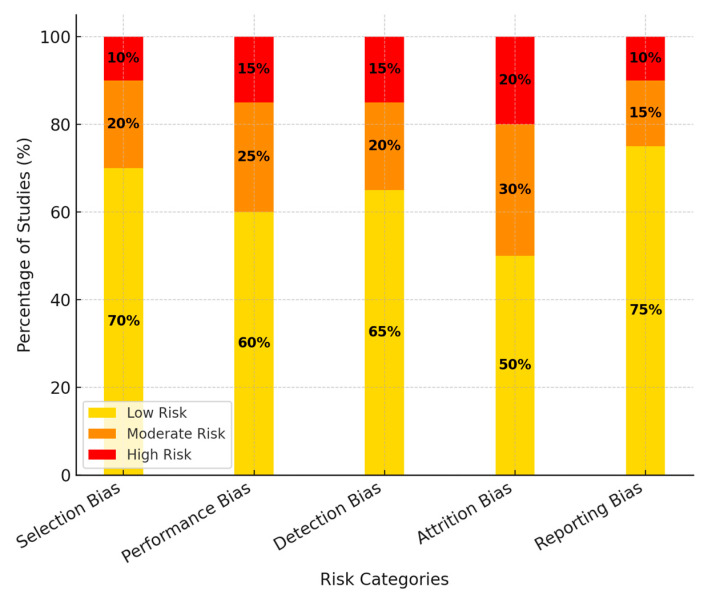
Risk of bias assessment across categories.

A detailed risk of bias analysis was performed using the Cochrane Risk of Bias Tool to assess the validity and reliability of the included studies. Selection, performance, detection, attrition, and reporting biases were evaluated in this review. Most studies had a low risk of randomization and allocation concealment. However, most studies did not report blinding of participants and personnel, which may have introduced performance bias. Attrition bias was observed in trials with a high dropout rate and incomplete data. However, some of these biases were overcome by applying an intention-to-treat analysis. Most studies reported outcomes corresponding to their statements of the protocol, indicating minimal reporting bias. Generally, although the studies included varied methodological rigor, sensitivity analyses did not exclude high-risk-of-bias studies to make the review’s findings robust. Figure 2 shows the distribution of risk levels (low, moderate, and high) across the five key bias domains: selection, performance, detection, attrition, and reporting bias.

## 4. Results

This systematic review identified 81 studies that tested the efficacy, feasibility, and applicability of NG-CBTs for depression, from mere digital tools and teletherapies to personalized treatments. It, therefore, considers various heterogeneous methodologies, including RCTs and other high-quality empirical research, which are eligible for inclusion in this comprehensive analysis of the effect of NG-CBT on mental health outcomes. These findings provide insight into how digital, and telehealth interventions improve accessibility and enhance therapeutic engagement, addressing some of the limitations of traditional CBT. The key findings are outlined below, organized to provide an overview of the effectiveness, feasibility, and challenges of integrating technological advancements within CBT frameworks.

### 4.1. [RQ1] How Can Technology, Such as Automated Text Messaging, Enhance the Delivery of CBT for Depression?

The automation of text messaging and digital CBT platforms has begun modernizing mental health care, particularly major depression, by increasing access for individuals who usually face barriers to care due to distance, mobility issues, or conflicting schedules, as identified in studies such as those by the studies [137,165,176]. These technologies are particularly beneficial for those in remote or underserved areas, offering a lifeline where conventional therapy is difficult to access [168]. The flexibility of dCBT, accessible via computers or mobile devices, allows patients to engage in therapy at their convenience, reducing the need to travel and thus accommodating personal schedules, a significant advantage noted in a study [176]. Richman, iCBT has several advantages extended, such as scalability-a crucial key in step-care that targets the community’s greater population. Digital interventions ensure a regular pace with several necessary reminders that most works point to being important for patients: to engage themselves and adhere to treatments by the studies [128] or [200]. Regular reminders and motivational messages from automated systems remind patients to engage in their treatment plans and homework. This finding is consistent with that of a previous study [127].

Continuing contact afforded by text messaging and e-mail sustains engagement, and daily push notifications from chatbots invite interaction with CBT content, as revealed in studies [157,179]. Further, such technological tools allow therapy personalization, that is, the tailoring of treatments to the specific needs of individual diagnosis and comorbidity, which often proves to be more effective than standardized treatment methods [157]. Personalization is further facilitated by automated feedback and support mechanisms, thus enhancing the therapeutic alliance and improving the outcome, as remarked by the study [187]. Platforms like Cue use continuous monitoring of patient behavior to deliver personalized “micro-interventions”, highlighting the potential of automated systems to tailor treatment dynamically [147]. The immediacy of automated text messaging enables real-time monitoring and feedback, which is crucial for making timely adjustments to therapy plans, as discussed in a previous study [160]. Systems like Woebot use natural language processing and machine learning to provide personalized conversations and psychoeducation and are representative of the depth of support technology can provide to individuals [179].

Digital tools allow continuous monitoring, enabling precise tracking of symptoms and behaviors that assist in effective intervention and the prevention of recurrence [168,183]. The advantages mentioned in the studies [173,192,203], have similarly in their research, is that automated text messaging vastly reduces the requirement for clinician time, hence considerably reducing the costs of CBT. Consequently, a reduction in therapist time will greatly reduce costs and make these interventions particularly valuable in resource-constrained settings. Digital interventions can be scaled up to reach large populations, which is crucial for addressing the global treatment gap for depression, especially in low-resource settings [194]. Integrating e-CBT with supervised care, the stepped-care model efficiently allocates resources and expands care capacity without compromising quality, as highlighted in a study [128]. Text messaging aids in structuring the therapy process by setting agendas, goals, and reminders, thereby reducing therapist drift from evidence-based practices [170]. It also maintains ongoing communication between patients and therapists, facilitating quick check-ins and support without needing full sessions, thus ensuring continuity of care [170]. Furthermore, automated systems can increase safety by monitoring language or symptoms such as suicidal ideation and providing crisis support [179]. Combining automated text messaging with other digital tools, like mobile apps or online platforms, develops a comprehensive digital health solution. The combination of several therapeutic elements increases the effectiveness of treatment, as illustrated by the RxWell app, which enhances mental health through a combination of health coaching and automated CBT techniques [191,194].

Moreover, they overcome significant obstacles to traditional therapies through considerations like cost, stigma, and geographical limitations; therefore, they are of unique value to the age groups under question [191]. Thus, automated support allows for less frequent and shorter therapy sessions, providing more manageable workloads for clinicians and more scalable therapy options [201]. The study [162] reported that 95% of adolescents liked going over text messages with their therapists. This shows how text messaging can help reinforce the concepts learned during therapy. Integrated text messages for homework reminders also lead to high user satisfaction. A study [188] demonstrated that the therapeutic alliance in videoconferencing CBT is comparable to that in face-to-face therapy, indicating technology’s ability to facilitate strong therapeutic relationships even when physical contact is absent. The study [172] noted that these increases in behavioral activation mediated decreases in depressive symptoms; thus, the automated messages effectively triggered and reinforced the therapeutic activities. Young adults also found the delivery format feasible and acceptable in CBT, which was delivered via text messages, and generally found high levels of engagement and acceptability in both conditions. Integrating these technological interventions enhances the delivery of CBT for depression by providing flexible, engaging, and cost-effective solutions tailored to individual needs and integrated with other digital health tools, ultimately improving the overall effectiveness of depression treatment [157,179].

Moreover, automated text messaging maintains therapeutic momentum of continued support utilizing timely interventions, which are critical in symptom management and relapse prevention, according to the study [181]. According to the survey, this is achieved by such statements of encouragement, reflections, and normalizing statements that make the patients feel supported [181]. The ability of automated systems to facilitate communication between patients and therapists allows for quick check-ins and support, which can help address issues without requiring full sessions [170]. This ensures continuity of care and structuring of the therapy process, such as agenda setting, goal setting, and reminders, which ensure that the therapy is focused and effective [170]. Moreover, regular monitoring of patients’ symptoms through automated text messaging provides real-time data that can be used to adjust treatment plans as needed, helping to identify any worsening of symptoms [181]. This capability enhances the overall accessibility of CBT, whereby patients can receive support from anywhere, which is particularly important in improving access to therapy for those who may face barriers [186]. This class of technology-powered interventions, including automated text messaging, is therefore cheaper than human-delivered therapy. They take a step toward reducing the overall cost of mental health care for consumers without sacrificing quality [184]. Systems provide personalized feedback regarding a patient’s progress or responses. This would translate into an advanced form of therapy, as treatments have been tailored to individuals [128]. Collecting and analyzing large amounts of data through technology can improve treatment outcomes by tracking patient progress using various metrics and informing treatment decisions [184]. It can reduce therapist time and, therefore, the costs of such treatments. Indeed, one study [173] found that a stepped-care program using Internet CBT with messaging support required less than half the therapist time needed for telephone-administered CBT alone.

Automated text messaging makes CBT more accessible to individuals who have difficulty attending in-person sessions due to geographical, physical, or time constraints, which is particularly beneficial for those in rural areas or with mobility issues [176]. It also helps maintain patient engagement and adherence to treatment protocols by providing regular reminders, motivational messages, and support [164]. Personalized automated text messaging provides feedback and support in personal ways, elaborating on the therapeutic alliance and the value of treatment outcomes. Continuous progress monitoring, together with ongoing feedback, facilitates rapid adjustments to treatment plans. Earlier, more responsive care [153]. The possibility of scaling up digital interventions, for example, automated text messaging far more efficiently than traditional approaches, might help bridge the global treatment gap for depression, particularly in low-resource settings where access to traditional therapy is limited [194]. Personalized feedback and reminders concerning specific treatment goals help sustain engagement and adherence to therapy [183]. Continuous support and real-time monitoring of symptoms are crucial for early intervention and adjustment of treatment plans, managing symptoms more effectively, and preventing relapses [183]. By reducing the need for frequent face-to-face sessions, automated text messaging makes CBT more cost-effective, lowering the overall cost of treatment while maintaining its effectiveness [164]. Making this whole therapy process active with the involvement of technology improves adherence to a treatment protocol because there is proof of the potency of digital CBT interventions across different demographics that tend to reduce depressive symptoms in the study [176]. Integrating automated text messaging with other digital tools, such as mobile apps and online platforms, provides a comprehensive digital health solution that enhances the overall effectiveness of treatment by combining various therapeutic elements [194]. Digital interventions, including automated text messaging, have helped overcome barriers to accessing traditional therapies, such as cost, stigma, and geographic limitations. These are particularly relevant for adolescents and young adults who face significant barriers to accessing mental health care [191]. Automated text messaging sends timely, personalized “micro-interventions” based on continuous monitoring of patient behavior, as in the Cue platform, which leverages smartphone data to provide customized interventions for improving social rhythm regularity [147].

Digital platforms using automated messaging maintain patient engagement through frequent, short skill-building techniques and motivational support, as demonstrated by the RxWell app (Version 1.9), which incorporates health coaching with automated CBT techniques to improve mental health outcomes [191]. Automated systems continuously monitor patient behavior and provide real-time feedback, allowing for more accurate and objective measurements of patient progress and more effective tailoring of interventions [147]. Digital interventions, including those using automated text messaging, are more cost-effective than traditional therapy, as found in the CoBalT trial, which showed that augmenting usual care with CBT was cost-effective for treating treatment-resistant depression [199]. Other digital interventions, such as automated text messaging, can be scaled to reach more of the population without a similar scaling of healthcare resources, meeting the widespread need for mental health services [178]. Automatic text messaging provides flexible automated support that supplements clinicians’ efforts. The fact that it could mean a time and frequency of the therapy sessions, hence less frequent, thus effective, helps in managing clinicians’ work and has made therapy more scalable [200]. Regular reminders, motivational messages, and prompts to practice CBT skills help maintain patient engagement and adherence to therapy, addressing the common issue of high dropout rates in stand-alone digital therapies [200]. Monitoring patient progress and providing real-time feedback enhances the therapeutic process by allowing timely treatment plan adjustments based on patient responses [167]. Personalized support and guidance help patients apply CBT techniques in their daily lives, which is particularly effective when combined with brief teletherapy sessions [200]. Integrating automated text messaging into CBT for depression improves accessibility, engagement, and cost-effectiveness while providing personalized and timely support to patients. Digital interventions like Internet-delivered CBT (iCBT) bridge the treatment gap in mental health by delivering scalable solutions accessible to a larger population, particularly useful in stepped-care models like the UK’s Improving Access to Psychological Therapies (IAPT) [182].

Continuous support and monitoring between sessions help maintain engagement and adherence to the treatment plan, which is crucial for effective results. Mobile technology with regular brief assessments can predict the development of psychopathology and support personalized health care [116]. Automated messaging interventions, as well as digital interventions in general, have proven to be cost-effective. At the same time, iCBT might achieve long-term cost-effectiveness in IAPT settings, which contributes to substantial improvements in depressive and anxiety symptoms [182]. In turn, this enhances the real-time collection of data gathered with automated text messaging and permits more accurate clinical decisions regarding their conditions, thus improving the intervention [116]. Mobile applications incorporating behavioral activation techniques can be used with ongoing therapy to enhance treatment outcomes, such as the Behavioral Activation app developed to support patients and therapists in implementing behavioral activation strategies. Digital interventions, whether supported by a lay coach or an automated system, are feasible and acceptable and lead to significant symptom reduction among diverse populations (even older age) with depressive symptoms and simultaneous improvement in anxiety and social isolation [204]. Automated text messaging can enhance CBT for depression by increasing accessibility, providing continuous support, improving cost-effectiveness, enabling real-time data collection, supporting behavioral activation, and being feasible and acceptable to users. This puts CBT within easy reach of people who could not have accessed it due to geographical, financial, or time barriers. It was noted that effective treatment via smartphone applications and videoconferencing has reached remote populations [168,189]. Continuous support and reminders between sessions through this therapeutic activity reinforce engagement; therapists use the back-end system to send personalized encouraging messages and weekly educational messages. This maintains the quality of treatment, reducing therapist time. Smartphone applications enable continuous monitoring of real-time symptoms and behaviors for more effective data-to-tailor treatment [168].

Technology decreases the overall treatment cost because there is no need to see patients as frequently, and blended treatment models are probably no less effective than complete face-to-face treatment but are more economical. Using more engaging digital tools is associated with better patient adherence to the treatment plan, and smartphone applications include databases of non-depressed behaviors, enabling participants to save and comment on their activities [168]. Automated text messaging and other digital tools offer flexibility, allowing patients to engage in therapeutic activities at their convenience, which is particularly beneficial for those with busy schedules or varying availability [168]. Regular reminders and motivational messages help patients stay engaged and adhere to the prescribed activities [160]. Automated systems deliver personalized feedback based on patient inputs, enhancing the therapeutic experience, with therapists providing asynchronous web-based feedback tailored to the patient’s progress and activities on platforms like Moodbuster [160]. The flexible and accessible delivery of CBT components makes it easier for patients to integrate therapy into their daily lives, which benefits those facing barriers to attending in-person sessions [206]. Real-time monitoring of symptoms and immediate support are facilitated through platforms like Moodbuster, which includes a mobile app for tracking progress and making timely adjustments to the treatment plan [160]. Automated text messaging makes CBT more scalable by reducing the need for constant therapist involvement, allowing more patients to receive timely and effective treatment [154].

### 4.2. [RQ2] What Are the Key Challenges in Integrating Digital Tools Within Traditional CBT, and How Can They Be Addressed?

Some of the challenges in integrating digital tools into traditional CBT limit the effectiveness and wide acceptance of such technologies. One of the significant issues surrounding digital interventions is how to provide regular and consistent user engagement. Recent research suggests that most digital mental health tools suffer from high dropout rates and inconsistent patterns of use. For example, a study [145] indicated that the major problem facing web-based CBT applications is poor adherence, with a median minimum completion rate of only 56%. In the same way, a pragmatic trial of online CBT packages, as indicated by the study [171], failed to show added value over usual care mainly because of low adherence rates. This issue is further supported by the study of [116], who noted that digital interventions without any form of human support yield considerably smaller effect sizes than those that contain even minimal support. Engagement challenges are also highlighted in a study [176] that reported attrition rates ranging from 50% to 83% in Internet-delivered psychotherapy, which suggests significant barriers to maintaining user participation. Furthermore, a study [170] indicated that poor compliance with homework assignments by the patient and low levels of engagement of the patient with digital tools can weaken the interventions. Including game-like and interactive designs may help to balance such issues. For example, a study [145] pointed out that participants interacting with a conversational agent used the tool 12.14 times on average over two weeks, showing higher engagement. In addition, the study [129] went further to suggest that therapeutic video game technology could also support high retention and consistent use among older adults with depression. In addition, the use of digital tools in addition to human support, such as therapist-delivered emails or personalized coaching, shows promise. For example, a study [157] pointed out that continuous contact with a therapist through e-mail helped participants remain engaged in treatment. Similarly, a study [204] showed that support from a layperson significantly enhanced adherence and overall outcomes of the Empower@Home program (A U.S. Department of Health and Human Services) for older adults. According to studies [128,170], engagement and adherence can also be facilitated through automated reminders, motivational messages, and interactive elements. One study [184] found that navigation coaching and regular text message reminders sustained patient engagement.

Accessibility and the usability of digital tools are essential, especially among a diverse population. Digital interventions are not accessible to all people, for example, older adults, those with low levels of digital literacy, and socioeconomically disadvantaged and rural groups. According to a study [159], problems with the usability of digital CBT programs among older adults can reduce adherence and treatment effectiveness. A study [128] noted that participation in e-CBT requires access to the Internet, and such access is consistent and reliable, which is not possible for everyone. The study [179] highlighted the importance of ensuring that digital interventions are accessible and used by diverse populations, including those from disadvantaged backgrounds. In addition, the development of user-friendly interfaces, such as large buttons and text, will facilitate use by older adults to a great extent [159]. Providing technical support and training can also enhance digital literacy and improve the usability of these tools [170]. Offering alternative formats, such as telephone-based support, can ensure that individuals with limited Internet access can still benefit from digital interventions [128]. Additionally, providing low-cost devices and Internet access can help overcome the financial barriers to technology adoption [203].

Another major challenge lies in maintaining a strong therapeutic alliance through digital formats. A review [188] found that the therapeutic alliance for videoconferencing CBT is comparable to face-to-face therapy, demonstrating that a strong therapeutic relationship can be maintained digitally. The shift to digital formats brings a great deal of consideration to how a sense of connection and support can be achieved. It may help if the treatment involves video calls and personalized feedback. According to a study, even reviewing text messages with therapists has proven helpful for teens [162]. Regular personalized communication can improve the therapeutic alliance, in which patients must feel supported and engaged. Researchers [190] also found no significant difference in working alliance scores between in-person and telehealth delivery, indicating that the therapeutic relationship can be preserved, if implemented appropriately. The integration of digital tools with more traditional face-to-face sessions, as discussed in a previous study [164], holds a balanced approach that capitalizes on the strengths of both modalities. In addition, researchers [187] reported that overall satisfaction and therapeutic alliance ratings were higher after an algorithm-based modular psychotherapy approach than after standard CBT, thus suggesting that digital tools support rather than replace human contact.

Personalization of digital interventions is considered an essential factor for their effectiveness. Digital tools are sometimes criticized for missing the personal touch of traditional face-to-face therapy. An effective, tailored Internet-based CBT was developed [157], which combined modules from different treatment packages based on the primary diagnosis and comorbidity. This involves developing digital tools that allow customization or tailoring to a patient’s individual needs to ensure that the treatment covers relevant issues for the user. According to a study [172], embedding mechanisms of change, like behavioral activation, in digital tools makes them much more effective. Again, he added that having personalized feedback and support options, just as with this text-message-delivered CBT, improves the effectiveness of such digital interventions. This can be further personalized by involving patients in the decision-making process and providing them with more choices regarding whether components are digital or traditional, based on their comfort and preference [160]. Safety and monitoring of potential risks, such as suicidal ideation, should be ensured in digital interventions. The study [179] added that the Woebot app has safety features to detect suicidal ideation and hotline numbers during a crisis. According to the same study [179], digital tools should have appropriate safety protocols, including real-time monitoring and automated warnings in the case of alarm behaviors or symptoms that would put a patient at risk. Crisis support systems like hotlines and emergency services should also be integrated into technology. Efficiency of Digital Tools Compared to Traditional Face-to-Face Therapy. Researchers in their study [202] noted that the combined CBM with iCBT resulted in significant reductions in both depressive symptoms and distress, suggesting that digital tools can be effective when combined with more traditional CBT components. Ensuring that digital tools are evidence-based and have demonstrated efficacy through rigorous testing, such as RCTs and meta-analyses, helps address concerns about their effectiveness [194]. For this to remain effective, treatment fidelity must be maintained for CBT to be delivered digitally; that is, the core components of CBT should be appropriately and consistently delivered using digital devices. Supervision becomes an essential ingredient of regular supervision and treatment manuals, allowing some latitude to individualize treatment according to specific patient needs, as stated by [165,198]. Moreover, digital tools must be continuously reappraised and readjusted for effectiveness based on clinical outcomes [141].

Technical problems in integrating digital tools include difficulties in the reliability of digital platforms. Software glitches, problems with Internet access, and platform navigation issues can affect treatment processes. One study [163] recorded the critical technical difficulties of using online platforms and Skype to conduct therapy. First, digital platforms must be reliable, secure, and user-friendly. Technical support and troubleshooting can be used to solve such issues [170]. Studies conducted on high-speed networks ensure that the connectivity during a videoconferencing session is reliable [189]. Other important factors include cost and scalability issues in digital interventions. While digital tools are promising for cost reduction and scalability, this should not occur at the expense of quality. Researchers [174] reported that therapists took much less time in a stepped-care program comprising Internet CBT with messaging support than in a preceding trial; hence, there was a significant difference in costs. Implementing a step-care model that begins with the least resource-intensive methods and moves to more intensive care based on patient needs can help allocate resources efficiently while maintaining high-quality care [128]. Utilizing Internet-delivered CBT (iCBT) as part of stepped care could enhance outcomes while managing costs [182]. Collaboration with industry partners and leveraging commercial platforms may ensure that digital tools are scalable and sustainable in clinical settings. As a study [200] argued, the seamless integration of digital tools into existing health systems is a significant challenge. This covers security and privacy issues and involves training healthcare professionals to use these tools. Researchers [171] have also mentioned that there should be a secure data exchange and that healthcare providers must monitor patient progress through digital platforms. Ensuring that digital tools comply with data protection regulations and implementing robust security measures may help alleviate concerns regarding data security [130]. A study [200] emphasized the necessity of incorporating active participation by patients and clinicians in the design process to ensure that digital tools are intuitive, engaging, and relevant to users’ needs. Indeed, comprehensive training programs and ongoing support for therapists can ensure comfort and proficiency with the technology [198]. The development of blended treatment models that combine online and face-to-face sessions can preserve the clinical effect while reducing the need for expensive face-to-face sessions [164].

Other, more general barriers to digital tool implementation include the acceptance of digital tools by therapists and patients. Therapists may be skeptical about the digital component or lack the skills to use it effectively. This issue can be addressed by properly training therapists, offering support, demonstrating the benefits of evidence-based digital tools, and creating an environment where therapists can share experiences and strategies. According to a study [160], patients are also likely to show variation in the use of digital tools, which affects the uniformity and effectiveness of treatment. Understanding patient characteristics and preferences and involving patients in decision-making can help to address these challenges [160]. Providing culturally tailored interventions and the option of initial in-person sessions can help build trust and rapport, addressing cultural barriers to accepting digital tools [143].

Treatment-as-usual heterogeneity may further complicate the estimation of the effectiveness of digital tools, as standard care practices can differ in various settings [191]. Standardization of treatment protocols and clinician training will reduce treatment heterogeneity. It is essential to mention that several quality assurance measures, such as standardized education and supervision of therapists, may ensure that care is delivered consistently [191]. Data security and privacy are vital when using digital tools for mental health. Ensuring that any digital platform with which a patient trusts their information satisfies the requirements for privacy codes and has superior security is essential [201]. Strong security and compliance with data protection regulations can help mitigate concerns associated with the confidentiality of patient data [130].

However, such complex issues require multivariate answers to make the incorporation of digital tools in traditional CBT more effective and beneficial for both patients and health systems. These include technological advantages, strategic and personalized treatment methods, extended support, accessibility, ease of use, and smooth integration into conventional medical practices.

### 4.3. [RQ3] What Are the Efficacy Outcomes of Teletherapy-Based CBT Compared to In-Person Therapy for Depression?

The efficacy results of CBT teletherapy against in-person therapy are quite reassuring; many studies report them to be just as effective in reducing depressive symptoms and greatly improving the quality of life of a subject. A recently published paper proved that the study [128] compared the effectiveness of electronically delivered versus in-person CBT for depression, and significant improvement in depressive symptoms and quality of life was established in both groups of treatment. This is further supported by a study [188] in which participants in both conditions, telehealth-delivered integrated CBT and usual care, evidenced a significant decrease in depressive symptoms over time, highlighting the feasibility and potential benefits of teletherapy. In a study [190], all groups in person or by telehealth reached significantly decreased Hamilton Rating Scale for Depression scores from pre- to post-treatment, suggesting no overall significant differences in effectiveness between in-person and telehealth modalities, with even better adherence in the telehealth group. The study [192] adds another dimension by showing that computer-assisted CBT using less therapist contact is highly cost-effective and maintains treatment efficacy, thereby reducing costs without any adverse outcomes. These findings suggest that teletherapy can be more compelling and efficient than in-person therapy. Researchers in their study [157] reported that both tailored and standardized Internet-based CBT exerted a significant effect on enhancing depression, anxiety, and quality of life, sustaining gains at the six-month follow-up, and remarkably, tailored ICBT was most effective for the more severely depressed comorbidity. The combined synergistic effect of cognitive-bias modification and iCBT was examined in a study [202], where the decrease in depressive symptoms and associated distress was significant, with a medium-to-large effect size; there was considerable improvement in secondary measures, such as disability, anxiety, and repetitive negative thinking.

Indeed, in one of the few recent studies [140], people with PD who underwent a personalized cognitive-behavioral telemedicine program recorded impressive gains in depression, anxiety, quality of life, sleep, negative thinking, and caregiver burden associated with both guided self-help and formal telephone-based psychotherapy. These studies document that teletherapy not only compares favorably in outcomes with traditional forms of CBT but also demonstrates efficacy across diverse populations and multiple domains of functioning. One study [158] also compared telephone-administered CBT and face-to-face CBT for major depression in primary care patients with alcohol use problems. Indeed, both conditions appeared to have similar treatment engagement and depression outcomes at all time points. The studies by [159] and [172] on, respectively, text-message-delivered CBT and Internet-delivered CBT facilitated by laypersons, coupled with their extension to older adults, young adults, and individuals with treatment-resistant depression, reported significant improvements in depressive symptoms. For instance, one study [172] found that young adults in the treatment group were three times more likely to have only minimal or mild symptoms of depression at two months compared with participants in the waitlist control. Researchers [171], in an RCT published in 2017 RCT, showed the efficacy of a smartphone CBT app as an adjunctive therapy, whereby patients on active app use scored significantly lower in depression assessments than patients on medication change alone, hence proving the potential of teletherapy-based CBT as an additional treatment. Other examples include the study [184], which investigated online mindfulness-based CBT in conjunction with usual psychiatric care and identified significant differences in depression outcomes compared to usual care alone; participants receiving conditions under CBT-M evolved significantly according to the inventory regarding depression. Another recently completed non-inferiority trial [184] tested online group CBT-M against in-person group CBT-M and found that online CBT-M might be a highly feasible, if not more cost-effective, option than standard therapy. The efficacy of teletherapy-based CBT compared to in-person therapy for depression was also highlighted in a study comparing home-based telehealth (HBT) delivery of Behavioral Activation and Therapeutic Exposure (BA-TE) for PTSD and major depression with in-person, clinic-based care; the results indicated that HBT was non-inferior to in-person therapy.

Another study evaluated the efficacy of telephone-administered CBT for depression in patients with Parkinson’s disease and found that this modality outperformed treatment as usual on all depression, anxiety, and quality of life measures, with significant improvements persisting over a 6-month follow-up period [141]. The additional work of the study [186] on the Sadness Program, an Internet-based CBT intervention, likewise showed significant efficacy in reducing depressive symptoms in American adults and presented large effect sizes for improvement within groups in a 10-week intervention period, further proving the substantial clinical improvements viable with teletherapy. Indeed, the study [144] on digital CBT for insomnia in pregnant women reported improvements in insomnia symptom severity and other secondary outcomes that were significantly better than those with standard treatment. Indeed, one study [155] showed that cognitive therapy and interpersonal psychotherapy yielded equivalent outcomes on average. However, there were individual responses indicating that teletherapy-based CBT, which includes cognitive therapy, could be as effective as in-person therapy for specific individuals. A randomized trial comparing brief interpersonal psychotherapy (IPT) and CBT delivered both in-person and via telehealth found that Hamilton Rating Scale for Depression (HRSD-17) scores declined significantly from pre- to post-treatment in all groups, with no significant differences between in-person and telehealth delivery, further suggesting that telehealth can be as effective as in-person therapy for reducing depression symptoms [190]. Indeed, a study [165] of telehealth CBT for insomnia co-occurring with depression in older adults revealed clinically significant improvements in both conditions maintained at a 2-month follow-up, further supporting the efficacy of teletherapy in treating depression within underserved populations. A study [148] reported that both sertraline and CBT, including teletherapy, resulted in remission of depression in over half of the patients with epilepsy and depression, with no significant difference in efficacy between teletherapy-based and in-person CBT.

Researchers in a study [173] examined telephone-administered CBT for veterans with major depressive disorder, using a high compliance rate and high therapist competence. It seems that teletherapy may have been effective under other conditions, but the specific population required a more arduous intervention. One study [194] compared disorder-specific CBT with transdiagnostic CBT, both delivered via the Internet. The significant symptom reductions that occurred in both major depressive disorder and comorbid anxiety disorders were without any marked differences across conditions, thereby showing evidence for the efficacy of teletherapy-based CBT in their treatment. In a non-inferiority trial designed by a study [174], a stepped-care program, including Internet CBT with telephone support, was compared to telephone-administered CBT, and the former was found to be non-inferior. This further establishes that teletherapy-based CBT is as effective as in-person therapy. However, patients who received telephone-administered CBT were more satisfied with their treatment. A study [176] tested a telehealth intervention for patients with uncontrolled diabetes and clinically significant depression; they concluded that, although there was no overall statistically significant improvement in symptoms of depression or glycemic control between the telehealth intervention and enhanced usual care, at 12 months, significantly more patients in the telehealth group achieved a depression response, which may indicate that, over time, teletherapy-based CBT could yield clinically meaningful improvements in depressive symptoms. Most demographic groups did not differ in their responses to the digital CBT interventions. In another recent randomized trial, digital CBT for insomnia resulted in clinically significant reductions in both insomnia and depression severity without substantial differences in efficacy across demographic groups [176]. Researchers in their study [174] investigated the effectiveness of an 8-week telephone-administered CBT for patients with multiple sclerosis and symptoms of depression. Results showed that depressive symptomatology diminished significantly more in the telephone CBT condition compared to the usual care control condition.

A meta-analysis of digital intervention debris, which includes teletherapy elements, showed a significant decrease in depressive symptoms. This effect size was as large as that observed for conventional face-to-face psychotherapy [194]. The study [196] demonstrated that guided self-help and individualized e-mail therapy are teletherapies based on the CBT format, which had significant symptom reductions with moderate to large effect sizes; a great percentage of the participants reached high-end-state functioning both at post-treatment and follow-up. Nicholas et al. 2019 supported the efficacy of Internet-based CBT within a stepped-care framework; this allowed evidence that CBT could be an effective low-intensity treatment for depression, while only a small proportion needed to be stepped up into the more intensive intervention. The study [147] showed that the Cue platform, a digital intervention based on social rhythm principles, demonstrated significant improvements in depressive symptoms over 16 weeks, especially in those with moderately severe to severe depression at baseline. A study [201] found that participants who received a smartphone-based CBT app in conjunction with brief teletherapy appointments had significant reductions in depression severity, with large effect sizes, which were maintained at a 3-month follow-up. Researchers in their study [152] indicated that digital CBT for insomnia was associated with a clinically significant improvement in both insomnia and depressive symptoms, with increased odds of clinically significant improvement in depressive symptoms at post-intervention and follow-up. A study [138] demonstrated that brief individual CBT administered in a primary care setting was more effective than usual treatment in terms of time to diagnostic recovery from major depression, with a significant benefit on most secondary outcomes during the first year of follow-up. The first pragmatic RCT within the UK Improving Access to Psychological Therapies (IAPT) program demonstrated a large improvement in symptoms of depression and anxiety using CBT. It stated that CBT is effective and probably cost-effective in the long run [182]. Empirical evidence also suggests that other supported digital interventions by lay coaches, such as the Empower@Home program among older adults, have also revealed significant efficacy, maintaining a medium effect size for depression reduction at post-test and follow-up [204].

The idea is that the effect sizes for Internet interventions without human support are substantially smaller than those with some level of human support; thus, CBT via teletherapy can be much more effective if supported to a certain degree [116]. In this regard, researchers in a study [189] conducted an RCT comparing CBT delivered via videoconference to in-person therapy for a mixed diagnostic cohort, including depression. They found significant reductions in symptoms of depression, anxiety, and stress, with improvements in quality of life in both treatment conditions and no significant differences between the teletherapy and in-person groups. Client and therapist ratings of the working alliance and client satisfaction were high for both conditions and did not differ significantly between the two modalities. This study [160] concluded that blended treatments, combining digital components with face-to-face therapy, appeared feasible and yielded promising results, with high treatment satisfaction and reduction in depressive symptoms. Similarly, combining online and face-to-face sessions was found to maintain clinical effects while potentially improving cost-effectiveness, indicating that teletherapy components can be integrated into traditional CBT without compromising efficacy [164]. Participants demonstrated significant reductions, compared with a waitlist, in the severity of their clinically significant diagnoses and self-reported symptoms of anxiety and depression, regardless of treatment delivery method, in person or via telehealth- [186]. The findings of this study point toward the potent efficacy of teletherapy-based CBT as an honest and effective option for in-person treatment of depression, offering advantages in accessibility, engagement, and adherence, where digital tools enhance these benefits.

### 4.4. [RQ4] How Can Teletherapy Delivery of CBT Be Optimized for Personalized Patient Care?

The teletherapy delivery of CBT can be significantly enhanced by providing personalized patient care by focusing on each patient’s unique needs and circumstances. One of the most important ways this can be done is by ensuring that the content of the therapy coincides explicitly with the patient’s diagnosis, symptoms, and personal background. For example, one study [157] mentioned that an individualized treatment plan should be established, combining modules from different treatment packages to ensure that the therapy is directly relevant to the patient’s primary diagnosis and any comorbidities. Similarly, a study [165] suggested that the presentation of manualized material be altered to accommodate the patient’s cognitive deficits. For instance, patients may require very simplistic language or visual aids may be utilized. In cases where patients exhibit high levels of depressive symptoms, even greater emphasis should be placed on this area of treatment. At this level of intervention, treatment is not a preordained construct but rather a very fluid and evolutionary process.

More flexible sequencing of therapy modules could allow even greater levels of personalization. For example, one study [160] delivered an intervention via the Moodbuster platform that allowed patients to proceed through the intervention at their own pace and in an order determined by themselves, thus accommodating individual preferences and enhancing engagement. According to a study [206], giving patients agency in their treatment process and providing them with decision-making opportunities improves adherence and satisfaction. Flexibility recognizes that every patient’s journey through therapy is different, and a rigid structure will not fit all. In addition to tailored content, regular and personalized feedback is crucial for optimizing teletherapy. The study [128] also established that the best approach is through weekly communication between the therapist and the patient through personalized feedback regarding the patient’s progress in the treatment. This constant feedback loop allows real-time adjustments in the treatment plan to keep the therapy aligned with the patient’s evolving needs. A study [171] further supported this approach in 2017 using a smartphone app, where patients could track their progress and receive individualized emails that reflected their activity level every week. Such personalized feedback may make patients feel supported and understood, thus enhancing the therapeutic alliance. Personalized feedback tools could improve mental health outcomes for patients while they await formal treatment, underlining the value of tailored support even before traditional therapy commences.

Digital tools are essential facilitators of real-time data collection and monitoring and are key constituents of personalized care. The study [184] utilized Fitbit step counts and online workbooks to monitor the patient’s progress, which helped him make evidence-based decisions regarding treatment. The studies [183,186] echoed that the importance of digital monitoring tools is that they provide early warnings about symptom worsening to enable timely intervention and adjustment in the treatment plan. These tools offer the therapist real-life glimpses into the daily experiences of the patient, allowing a better and more nuanced understanding of their condition, thus making for more precise and effective interventions. A study [168] demonstrated how therapists could use smartphone applications to monitor daily activities and mood and make treatment plan adjustments in real-time to make the therapy responsive and dynamic. The integration of easy-to-use platforms will ensure maximum patient involvement and adherence. In addition, the study [203] stated that to make teletherapy more accessible, there is a need for devices at low cost, Internet access, and easy-to-use interfaces. The study [170] noted the need for platforms that are easy to navigate and training for patients with limited experience with computers. Technology can be an enabler and does not create barriers to effective therapy. According to a study [164], blended care that includes web-based components may have better patient participation because of the assured high usability of the system. In addition, a study [168] mentioned a very user-friendly smartphone application because participants could easily log activities or interact with the program; hence, a thoughtful design has considerable potential to improve engagement significantly.

Other significant ways of optimizing teletherapy include enhancing communication. Furthermore, a study [188] established that videoconferencing and face-to-face therapy worked because they maintained a good therapeutic relationship. In addition, the study [163] called for multiple modes of communication, such as telephone, online chat, and video calling, to meet patient preferences for modality and, by extension, to enhance engagement. For instance, automated messaging, discussed in studies [170,199], could provide regular reminders, support, and encouragement that might facilitate continuity of care. For example, a study [172] found that text message-delivered CBT was more effective for the young adult population because it was consistently delivered and very engaging. Communication may occur through messaging, video, voice, and chat. In this way, patients may use a medium that better fits their lifestyle or comfort zone to enrich their interaction with therapy. Generally, digital interventions are made highly effective with the help of human support, whether a lay coach or a professional therapist. For example, the study [204] revealed that Empower@Home, including the support of lay coaches, showed surprising reductions in depressive symptoms. Similarly, a study [116] found that professional coaching embedded in digital interventions was associated with improved adherence and better outcomes. In addition, a study [159] described the delivery of layperson-facilitated Internet-delivered CBT, with weekly check-ins with a supportive guide addressing potential failure points and providing personalized support. The human touch bridges the chasm between cold digital tools and personal interaction that helps patients by offering encouragement and a sense of accountability, which is necessary for progression in their therapy.

Natural language processing and machine learning are state-of-the-art technologies for extending teletherapy personalization. The study [179] introduced the Woebot app, which leverages these technologies to offer personalized conversations and CBT-based psychoeducation tailored to the individual’s reported mood or needs. This dynamic interaction thus enables a more responsive and adaptive therapeutic experience. Additionally, researchers in their study [181] discussed the use of machine learning in developing personalized intervention rules for co-occurring conditions, such as insomnia and depression, further providing a technological scope for personalizing interventions with better precision. Similarly, ensuring safety features and addressing ethical concerns are integral parts of personalized teletherapy. In addition, a study [179] identified that the Woebot app contains safety features that detect suicidal ideation and provide hotlines for crisis situations. Moreover, the study [191] integrated risk assessment protocols into the RxWell app for monitoring patient distress and guaranteeing timely interventions, underlining the importance of assuring the safety of patients within digital interventions. In addition, the study [130] added that data security issues should be treated in correspondence with regulations on data protection to increase trust and promote the use of teletherapy.

Accessibility is also essential, and teletherapy needs to be accessible through many platforms and for people with different levels of digital literacy. Nicol (2022) emphasized how digital interventions must be even more user-centered and accessible to diverse populations, including socioeconomically deprived and rural communities. Facilitating flexible accessibility to therapy means that more types of patients are reached, and access to personalized treatments is extended. Furthermore, ongoing training and support for therapists themselves optimize the delivery of teletherapy. The study [198] thus stressed the training and supervision of therapists to render them comfortable and confident with the technology. Reiterating these views, one study [167] stated that continuous training helps therapists integrate digital tools into their practice. In addition, the study [189] discussed that continuous supervision with fidelity to protocols is important in maintaining quality. This will go a long way in ensuring that the quality of care is improved, where the therapists are confident in providing personalized interventions. Therefore, it is paramount that comprehensive training and support be provided to therapists in order to use teletherapy platforms for the personalization of care. Training should address not only the technical use of digital tools but also how to maintain a therapeutic alliance in a virtual setting. Additionally, the study [198] emphasized that training and supervision of therapists should help them become comfortable and proficient with technology. In a related vein, the study [167] emphasized that such training is an ongoing process for continuous support for therapists in integrating digital tools into their work. A previous study [189] stated that continuous supervision and adherence to the treatment protocol are essential for quality assurance. In a similar vein, the study [160] suggests ongoing support and supervision to allow the therapist to offer care to a patient on an individual basis. The truth is that with therapists who are well-prepared for digital tools and are thus confident enough to give personalized interventions, quality may greatly improve in such instances.

Blended treatment approaches, which merge digital tools with traditional face-to-face therapy, are another avenue for enhancing personalized care. A study [116] indicated that blended treatments allow for more efficient care in that patients who can manage their mood independently are able to do so, reserving in-person resources for those in greater need. In addition, a study [164] found that blended CBT, which alternates between online and face-to-face sessions, maintained clinical effects while potentially improving cost-effectiveness. Additionally, a study [168] showed that blended treatment using a smartphone application, and four face-to-face sessions were effective compared to full, ten-session, face-to-face therapy but with considerably less therapy time. This underlines the flexibility of blended treatment, which allows for personalized care as patients needs change easily and much more efficiently than otherwise. In addition, both cost-effectiveness and scalability must be considered for teletherapy. Further, a study [182] showed that Internet-delivered CBT (iCBT) could be cost-effective in the long term within the UK’s IAPT settings. By reducing the overall cost of therapy, personalized care becomes more accessible to a wider range of patients. Additionally, a study [168] found that a blended treatment approach could reduce therapist time by 47%, thereby lowering costs and making therapy more scalable. Developing interventions that can easily scale larger populations while maintaining personalization in mental health services is vitally important. According to researchers [201], digital tools offer great opportunities for improving clinicians’ workload, which in turn makes therapies more scalable using modern technology.

Furthermore, a study [147] delivered personalized interventions through the Cue platform based on continuous monitoring, which may be effective, further supporting the value of content personalization in therapy. This can be considerably extended in terms of making care more personalized through platforms that provide personalized “micro-interventions” based on continuous monitoring. These micro-interventions are small, focused interventions tailored to specific behaviors or symptoms at the moment and provide just-in-time support that is highly relevant to the immediate needs of the patient. Real-time data from digital tools allow therapists to deliver such interventions precisely at the point of need. Continuous monitoring of the progress of patients and timely feedback are important components of personalized care. According to a study [160], therapists operating the Moodbuster platform checked patient progress and provided written feedback individually. Similarly, it is possible to fine-tune treatments continuously to meet the needs of patients. This continuous follow-up allows for changes in treatment where necessary, thus keeping the therapies effective. This included the Moodbuster mobile app for daily mood monitoring, allowing great insight into the status of the patient for timely intervention. This study demonstrated the usefulness of real-time data in making treatment decisions that better match a patient’s current state. Greater patient involvement in decision-making with respect to treatment might further enhance personalization. One study [206] argued that a shared decision-making process between the patient and therapist may allow consideration of the patient’s preferences and needs. It is a collaborative approach wherein the treatment is developed to meet the goals and values of the patient; hence, it is better aligned to ensure satisfaction and adherence. Person-centered and more effective care will result from therapists being more involved with their patients.

Optimal teletherapy requires proper training for therapists to use digital tools and integrate them into their practice. Continued support and supervision enable therapists to individualize care for the patient, as highlighted in the study [160]. Further, this training and support ensure that therapists are confident in using technology as a tool to enhance care for better treatment experiences among patients. These include tailoring content, regular and personalized feedback, advanced technologies, ensuring safety and accessibility, and offering flexible methods of communication. With all these, the teletherapy delivery of CBT can be optimized to highly individualize patient care. This comprehensive approach ensures that the treatment is responsive to individual needs, leading to improved engagement, adherence, and overall treatment outcomes. Integrating all these elements presents a dynamic, adaptive, and patient-centered therapeutic experience that maximizes benefits for everyone.

### 4.5. [RQ5] How Can CBT Be Personalized Using Digital Tools to Improve Both the Cost-Effectiveness and Accessibility for Patients with Depression?

CBT can be significantly modified with the strategic use of digital tools to make it more accessible and affordable. One powerful way to do this is to develop modular treatment plans that are then tailored to the individual patient’s needs based on their primary diagnosis, any co-occurring conditions, and personal preferences. The findings of the study [157] presented that such a combination of modules from different treatment packages separately would render the therapy highly relevant and might also minimize the necessity for more intensive and expensive interventions. Other systems use simulated dialogues to set the content dynamic dependent on user responses, a factor referred to by the study [194] that considerably raises user engagement and the general effectiveness of treatment. The app Woebot, as introduced in studies [145,179], applies natural language processing and machine learning for personalized conversations or CBT-based psychoeducation based on self-reported mood or current needs. This degree of dynamic adjustment of content makes the therapy relevant and interesting over a longer period. The PAI approach, according to a study [155], utilizes baseline predictors and moderators to make highly individualized treatment recommendations, thus optimizing resource allocation and improving patient outcomes. Similarly, a study [187] outlines the advantages of algorithm-based modular psychotherapy, which offers flexibility and a personalized treatment approach by adapting transdiagnostic modules to the patient’s individual profile, thus leading to higher overall satisfaction and a stronger therapeutic alliance.

Some important elements in any therapy that can be significantly enhanced by various digital strategies include engagement and adherence. In addition, the studies [127,172,184] have all reported the benefits of frequent automated text messages, which help enhance engagement and increase attendance at therapy to maintain participation in CBT and reduce dropout rates. According to a study [128,171], personalized feedback plays a very important role in making feedback pertinent to the progress of patients and thus achieving better mental health by meeting their needs and preferences. In addition, a study [183] cited the role of personalized ESM. Symptom and behavior monitoring in real-time can enable the personalization of feedback and real-time adjustments of treatment strategies, thus allowing for effective symptom management and prevention of relapse. Moreover, the use of interactive features such as gamification, as described in a study [129], and even multimedia elements like videos or interactive exercises, studied by [161], make the process more interesting and provide better learning. Digital tools offer unparalleled flexibility in the delivery of CBT. Availability through multiple modalities, including the telephone, online platforms, and video calls, means that the patient will prefer the modality they feel comfortable with; levels of engagement and adherence have increased [163,188]. As studies [137,143,176,186] have pointed out, the possibility of undergoing therapy from the comfort of one’s own home reduces the need to travel to a therapist and accommodates mobility issues or people living in remote areas. It also enables blended care models, where face-to-face treatment is combined with web-based components, to further improve accessibility, reduce treatment duration, and lower costs overall because clients can work through part of the treatment protocol on their own, as discussed in study [164].

One of the biggest advantages of digital CBT is cost savings. The stepped-care models described in the studies [128,174] start with less intensive digital interventions, increasing to more intensive treatments only, when necessary, thereby optimizing resource use and reducing the extent of therapist time. The study [192] shows that computer-assisted CBT significantly reduces therapist contact while preserving efficacy, lowering costs, and increasing accessibility. Asynchronous communication, in which therapists provide feedback and support at different times, further optimizes therapists’ use of time and resources. This was indicated in a previous study [128]. Structured telehealth interventions, such as those focused on collaborative goal setting and behavioral activation, as described in a previous study [176], allow for more personalized and accessible services at a lower cost. Digital tools provide structured, evidence-based interventions that can be accessed from patients’ homes, reducing the need to travel long distances and, thus, reducing costs [186]. Automated reminders and support keep individuals engaged and compliant, which improves outcomes. Automated text messaging and digital communication facilitate ongoing support and quick check-ins between sessions, continuing care without additional in-person visits, as noted in a study [170].

Digital tools also break many barriers to conventional therapy and promote inclusiveness in health care. In improving digital literacy, along with access to low-cost devices and the Internet, teletherapy may have a chance to become more inclusive for the populations of low-income status, according to the proposal of the study [204]. Making these platforms user-friendly and accessible, as reiterated in the studies [164,170,179] raises the level of treatment engagement and adherence among patients, even when their levels of digital literacy may differ. Accommodating age-friendly interfaces in the case of digital CBT, which is designed for homebound older adults or, alternatively, population-specific themes-for example, the study’s [159] work among pregnant women and the study’s [161] advances accessibility and participation. According to the same study [159], adding support from either specially trained laypeople or coaches in digital CBT might offer personalized support for failing points and, hence, be more effective. Strong safety policies, such as tracking suicidal ideation and crisis hotline numbers, remain crucial for monitoring the welfare of patients, as highlighted by this study [179].

Continuous monitoring and improvement are key to the success of digital CBT. Real-time data on patient symptoms, collected from digital tools, enable timely adjustment of the treatment plan and the provision of timely intervention in case of worsening symptoms, as identified by the studies [183,186]. As a matter of fact, such data collection and analysis of patient progress will enable the treatment plan to be adjusted in real-time and ensure that resources are used efficiently, according to the study [184]. According to a study [198], training and support for therapists will ensure that they can incorporate digital platforms to offer high-quality, personalized care. In addition, pre-structured content and resources that guide therapy sessions, as well as homework, keep therapies focused, curtailing therapist drift from evidence-based CBT through digital tools [170]. Digital platforms, which offer flexibility in scheduling, can be most helpful in accommodating the different needs and availability of patients to regularly attend sessions and avoid dropout rates [165]. A combination of such strategies sets forth the transformative potential of digital tools in the personalization of CBT, turning it into a more effective, accessible, and cost-efficient option for treating people affected by depression. It enables tailoring, increases engagement, allows flexible delivery, reduces costs, and is inclusive; therefore, digital CBT holds great promise for the treatment of mental health.

### 4.6. [RQ6] What Role Do Mobile Apps and Digital Platforms Play in Delivering Next-Generation CBT for Depression?

The integration of mobile apps and digital platforms into the CBT arena represents a step in the evolution of service delivery that will go beyond convenience to enhance the core therapeutic process of managing depression. This digital transition tackles some well-known hurdles that traditional therapy has faced for quite a while: accessibility, irregular patient compliance, and the need for more personalized and evidence-based treatments. According to the study [179], the study shows that mHealth interventions can unobtrusively pass into the daily routine of adolescents; it also represents how easily they are likely to access care for their mental health, something important at times of quarantine or workforce shortages. What makes these tools particularly effective is their capability for remote therapy, especially in rural or underserved areas where mental health professionals are scarce, a fact well documented in the studies [165,186]. Remote accessibility further helps individuals with mobility impairments or those whose work or personal commitments make attending in-person therapy difficult. Such platforms, as discussed in a study [188], further enhance the accessibility and flexibility of treatment by offering video calls, phone calls, and text messaging to communicate, thus being able to address a wider range of patients’ needs and preferences.

Moreover, these digital platforms are designed to encourage and maintain active patient involvement, which is a crucial component of any therapeutic approach. They do this through various features incorporated, such as automated reminders to patients of their upcoming therapy sessions and completion of tasks at hand, based on studies [128,170]. The introduction of gamification, interaction with multimedia content with videos and exercises, and even simulated dialogues make treatment more dynamic and engaging, hence increasing the patient’s motivation and adherence to therapy. This is supported by previous studies [129,161,172]. Continuous support mechanisms, like daily check-ins and readily available resources, would keep patients in touch and supported throughout their treatment journey, as discussed in previous studies [179,184]. Continuous contact with the therapist through e-mail helped participants stay engaged in the treatment, indicating the importance of ongoing support [157]. It is also important to note that although these platforms reduce the need for constant interaction by the therapist, some support from the therapist in the form of check-ins or personalized feedback immensely enhances adherence and engagement, as documented in a study [203].

Personalization capabilities represent serious developments in digital CBT platforms toward the personalization of treatment. These platforms can dynamically adapt the content, pace, and focus of therapy in response to patient responses, preferences, and progress, as supported by research [130,155,159]. According to a study [155], individual treatment recommendations for inclusion in digital platforms could be made so that treatments become more tailored to fit specific patient needs and thus maximize treatment success. This level of personalization is further extended by the fact that therapists use data collected by these apps to provide extremely individualized feedback and modify treatment plans in real-time, a practice adopted by the studies [128,160,171]. For example, the PAI approach, which integrates baseline predictors and moderators to generate personalized treatment recommendations, has been proposed [155]. Platforms like the Cue platform use data from smartphones to provide timely, personalized “micro-interventions”, some of which are aimed at improving regularity in social rhythms, highlighting the ability for care to be very individualized and proactive, a point also made by the study [147]. A combination of health coaching along with the use of digital CBT platforms, as in the RxWell app, further enhances personalization and reinforces the principles of CBT while offering motivational support, with reportedly tremendous results in improving mental health outcomes, as reported in a previous study [191]. Tailored Internet-based CBT, which combines modules from different treatment packages depending on the patient’s primary diagnosis and comorbidity, is sometimes more effective than the usual standardized approaches [157].

In sum, digital delivery of CBT is cost-effective from an economic point of view: costs attributed to mental health, in general, are considerably reduced because, first, there is less direct contact; second, therapists could see more patients and shorten treatment times, thus saving money; and this all agrees with statements by the studies [128,143]. In contrast, blended treatment models have demonstrated an increase in cost-effectiveness, combining the strengths of digital tools with conventional face-to-face sessions while maintaining positive clinical outcomes, as mentioned in a previous study [164]. In applications such as Good Days Ahead, computer-assisted CBT yields results comparable to those seen with traditional CBT, with only a fraction of therapist contact and, therefore, significant cost savings [192]. The CoBalT trial showed that CBT plus usual care was cost-effective for treating treatment-resistant depression [199]. Digital interventions could be scaled for more population impact without an accompanying increase in healthcare resources; this has important implications for providing mental health services at the population level [178].

Another important advantage of the scalability of digital platforms is that it has a great capacity to reach an enormously larger population, hence addressing the global treatment gap in depression, especially in low-resource settings, identified by the studies [128,194]. The platforms are also very effective in channeling resources and increasing care capacity without compromising quality, according to the studies [128,178]. The ability to deliver therapy to many individuals simultaneously reduces the burden on healthcare providers and makes mental health care more widely available [128]. Moreover, mobile apps and digital platforms facilitate continuous monitoring of symptoms and progress, as outlined in previous studies [176,183,186]. This real-time collection of data allows for the possibility of early intervention, enables timely adjustments in treatment plans, and helps predict problems that might arise, according to the studies [116,160,184]. Thus, the Moodbuster platform, a mobile application for daily mood monitoring, allows patients and therapists to track progress, enabling timely adjustments and making treatment effective, as seen in a study [160]. These innovative solutions include Woebot, a conversational agent delivering CBT content, for which a study [145] reported significant reductions in depression symptoms. According to the study, the Online Psychotherapy Tool (OPTT) is an e-CBT platform supported by a therapist that allows for personalized feedback and structured program delivery [128]. Other tools, such as behavioral activation and support strategies of behavioral activation in concert with ongoing therapy, are discussed in more detail, while through a smartphone, one can access CBT-sourced content and some practices available on Mindset for Depression [201]. Digital interventions can be complemented with short virtual therapy sessions and personalized help. For instance, a mobile CBT app that incorporates short videoconferencing with a therapist could provide personalized support, monitor improvement, and allow treatment adjustments to meet the specific needs of everyone [201].

Moreover, these digital aids also prevent the therapist from drifting through pre-structured content and resources guiding therapy sessions and homework assignments, enabling the therapist to stay focused and conform to evidence-based practices, as stated in the study [170]. They also incorporate advanced machine learning techniques in developing personalized intervention rules for prescribing the optimal digital treatment, especially for comorbid conditions such as insomnia and depression, as the study underlines [181]. Poor digital literacy can be overcome through the assurance that digital tools will be user-friendly, and technical support will be provided to overcome those barriers, enhancing the overall user experience and assuring that patients can benefit from digital CBT, as stated in the study [194]. Finally, the incorporation of mobile apps and digital platforms into CBT for depression is a paradigm shift in mental health. Emerging technologies represent a combination of greater accessibility, enhanced engagement, personalized interventions, cost-effectiveness, and scalability. In addition to increasing the efficiency and effectiveness of mental health care, it addresses the treatment gap and improves the life experiences of people with depression. These digital tools will finally move the field of mental health care toward a more patient-centered, data-driven, and accessible future.

### 4.7. [RQ7] How Effective Are Current Digital CBT Tools in Providing Long-Term Treatment Outcomes for Depression?

Current research provides a promising outlook on the effectiveness of digital CBT tools in achieving long-term positive outcomes for individuals experiencing depression. Indeed, several studies have documented sustained improvements in depressive symptoms, overall quality of life, and other related measures. These improvements often persist for extended periods, ranging from several months to a year or even longer. For example, one study [192] reviewed the effectiveness of a CCBT course called Good Days Ahead. It was determined that participants further improved at both the three- and six-month follow-ups, with less than a 10 percent relapse rate for those who achieved remission. Researchers in their study [157] investigated both tailored and unadopted Internet-based CBT (ICBT); large effect sizes were recorded for variables like depression, anxiety, and overall quality of life. At the 6-month follow-up, the gains were maintained. Researchers in their study [196] compared guided self-help with individualized e-mail therapy. Both treatments resulted in significant symptom reductions, and a large percentage of the participants, 47.4% for the guided self-help and 43.3% for the e-mail therapy, reached the criteria for high-end-state functioning at the six-month follow-up. These findings strongly indicate the potential of digital interventions to bring about long-lasting positive changes in the welfare of individuals. Digital CBT is considered durable. Another study [171] endeavored in the same direction. In this article, the authors explored a smartphone CBT app administered to individuals with antidepressant-resistant major depression. Surprisingly, the app’s effects continued until week 17, proving that it can sustain its effect for a considerable period. Further supporting these findings, a study [186] investigated another variant of iCBT, the Sadness Program. This treatment was associated with significant symptom reductions, large effect sizes, and strong clinical response and remission rates over the 10-week intervention period. Concerning the integration of digital technologies into more traditional modes of treatment, one study [170] reported significant and stable changes across measures of anxiety and depression. These gains were maintained even at the 12-month follow-up in a study that combined Internet-based support systems with face-to-face CBT sessions. The CoBalT trial, one of the most extensive studies in this area, further added to the growing evidence of the long-term efficacy of digital interventions. As Wiles (2014) reported, enhanced usual care plus CBT reduced depressive symptoms, and these benefits were sustained over the 12-month duration of the study. These results are promising, and more recent studies have continued to reinforce these findings. Researchers [182] conducted a study within the UK’s Improving Access to Psychological Therapies (IAPT) program. They found that iCBT not only improved depressive symptoms but also continued to improve up to 12 months post-treatment. In addition, researchers [168] examined a blended treatment model in which participants used a smartphone application in conjunction with face-to-face sessions. This approach naturally resulted in sizable within-group effect sizes and high recovery rates, which were impressively sustained at follow-up assessments.

Similar studies on new digital CBT show its efficacy compared to traditional therapy sessions. Additionally, researchers [128] conducted a study in 2023 on an e-CBT program supported by a therapist for patients diagnosed with MDD. The researchers found that this online program brought improvement in the symptoms of depression and quality of life, which was not different from in-person therapy. Moreover, a study [190] further supported these findings, reporting that both briefer interpersonal psychotherapy and CBT delivered via telehealth were just as effective as in-person therapy in reducing depression scores, showing the continued benefits of telehealth interventions. Researchers in their study [148] also did not find any significant difference in the effectiveness of teletherapy-based CBT compared to in-person therapy when treating patients with epilepsy and depression. Another study [174] compared the results of a step-care approach involving Internet CBT with telephone support to CBT alone. According to the study, the findings showed that the long-term effects of the step-care approach were similar to those seen in CBT. Stubbings (2013) also did not find any significant differences between the teletherapy and in-person groups in symptoms of depression, with clinical gains being maintained at follow-up. It was suggested that the delivery format does not significantly impact the therapy’s effectiveness. Digital CBT has also been shown to be effective in specific populations. The results of a study [188] indicated that symptoms were maintained at a three-month follow-up for integrated telehealth-delivered CBT for depression and insomnia in rural older adults. Researchers in their study [140] reported the following benefits for patients with Parkinson’s disease because of the telemedicine program: reduction in depressive symptoms, anxiety, improvements in quality of life, sleep, negative thoughts, and caregiver burden; these gains were observed for 4 months. In a study [176], dCBT-I not only significantly reduced the severity of depression at 1-year follow-up but also reduced the incidence of moderate-to-severe depression by half in those who initially had minimal to no depression. Dobkin also reported that T-CBT outperformed TAU on depression, anxiety, and quality of life measures in patients with Parkinson’s disease, with gains maintained over a 6-month follow-up. In a recent study [144], digital CBT-I among pregnant women was associated with significant and sustained improvements in insomnia and related outcomes. A study [152] proved that digital CBT for insomnia was associated with substantial improvements in both insomnia and depressive symptoms, indicating a strong connection between sleep improvement and long-term depression outcomes. These findings were further supported by a study [144] that established that digital CBT-I administered during pregnancy ensured enduring benefits for postpartum insomnia, depression, and anxiety at 3- and 6-month follow-ups.

Researchers in their study [179] thus provided some preliminary evidence that the natural language processing and machine learning-powered Woebot app may have sustained effectiveness in adolescents with depression and anxiety. Researchers in their study [184] also reported that an online mindfulness-based CBT (CBT-M) intervention added to usual psychiatric care led to significant improvements in depression outcomes with high retention, suggesting possible long-term benefits. In the short to medium term, a study [172] reported that a CBT-txt intervention significantly improved depressive symptoms, with particular benefit at 1-month follow-up. Additionally, a study [165] found that telehealth CBT for older adults with co-occurring insomnia and depression produced clinically significant improvements, which were well-maintained at the 2-month follow-up. According to a study [147], the Cue platform is based on continuous monitoring of patient behavior and delivers personalized “micro-interventions”. Their study indeed showed significant improvements in depressive symptoms over 16 weeks, especially among those with moderate-to-severe depression at baseline. In addition, a study [199] reported that the Mindset for Depression app, combined with brief teletherapy appointments, resulted in significant decreases in major depression severity that were maintained at a 3-month follow-up. The Empower@Home program, tested in a study [204], showed medium-to-large effect sizes in depression reduction at post-test, with sustained effects at a 10-week follow-up. In addition, a study [161] emphasized that such computer-assisted CBT interventions should be tailored to ensure acceptability and effectiveness across diverse groups, including pregnant women. A study currently underway [159] tests the effectiveness and feasibility of Internet-delivered CBT facilitated by laypersons among homebound older adults, and preliminary results look promising. The study [128] also discussed the stepped-care model of e-CBT with supervised care, which may ensure a more enduring long-term outcome by allocating resources at their best. On the other hand, one study [193] noted that both clinician-guided and self-guided formats of Internet-delivered CBT resulted in large reductions in symptoms of major depressive disorder and comorbid anxiety disorders. Regarding specifically uncontrolled diabetes and clinically significant depression in the sample, one investigation found large and sustained symptom reduction through telehealth. According to a study [164], there may still be more room for shorter treatments and highly valued long-term results through blended approaches to CBT for patients suffering from the most severe states. A meta-analysis of a study [194] on digital intervention debris revealed significant reductions in depressive symptoms, with effects increasing from three to six months post-treatment. Researchers in their study [183] stressed that continuous support and real-time monitoring are required to guarantee long-term engagement in and adherence to digital interventions. Additionally, a study [116] suggested that, in general, digital interventions that include human support will have better long-term outcomes than those that include no human contact. In addition, the study [181] added that developing individual intervention rules for prescribing digital treatments might improve long-term outcomes by allowing individualized interventions. A closer look at the effectiveness of digital interventions investigated in the study [196] showed that guided self-help and individualized e-mail therapy produced considerable symptom reductions with moderate to large effect sizes. Many subjects in both conditions achieved high-end-state functioning at the six-month follow-up, indicating sustained improvement. Researchers in their study [199] also reported the CoBalT trial, which added to the evidence base demonstrating the long-term effectiveness of digital CBT. In this study, augmenting usual care with CBT produced sustained reductions in depressive symptoms over 12 months. The Cue platform [147] utilized personalized “micro-interventions” based on continuous monitoring of patient behavior, resulting in significant improvements in depressive symptoms over 16 weeks, particularly in those with moderately severe to severe depression at baseline. Indeed, a recent investigation [201] found that participants receiving the Mindset for Depression app with added brief teletherapy achieved significant reductions in depression severity maintained at 3-month follow-up. A study [152] reported that digital CBT for insomnia, which also addressed depressive symptoms, significantly improved both insomnia and depressive symptoms; improvements in insomnia at mid-intervention mediated a significant portion of the effects on depressive symptoms at post-intervention. A study [144] presented findings that, when administered during pregnancy, digital CBT for insomnia yielded durable benefits for postpartum insomnia remission and significant improvements in depressive and anxiety symptoms at 3 and 6 months postpartum.

The long-term benefits of iCBT have been highlighted in various studies. A pragmatic RCT performed within the UK IAPT by the study [182] compared outcomes with iCBT, concluding that it significantly improved depressive and anxiety symptoms; indeed, further improvement continued for up to 12 months. The Empower@Home program’s nine-session remote intervention with a lay showed, according to the study [204], a medium-to-large effect size in a reduction in depression both at post-test and at the 10-week follow-up. Digital interventions with human support generally show better long-term outcomes [116]. This suggests that adding professional coaching or layperson support may enhance the long-term efficacy of digital CBT tools. Furthermore, digital CBT tools that use advanced machine learning methods to develop individualized intervention rules can offer personalized treatments that may improve long-term outcomes. For example, the study [181] outlined a protocol aimed at developing and evaluating an individualized intervention rule for prescribing the optimal digital treatment for co-occurring insomnia and depression, which may lead to better long-term outcomes. In addition, a study [168] compared a blended treatment approach smartphone application plus four face-to-face sessions, a complete ten-session face-to-face treatment, and found significant improvements in both groups over time on the primary outcome measure, BDI-II, with sizable within-group effect sizes and high recovery rates maintained at follow-up. One study [189] compared CBT delivered via videoconference versus in-person therapy for a mixed diagnostic cohort, including depression. Symptoms of depression, anxiety, and stress were significantly reduced, and quality of life improved in both conditions. There were no significant differences between the teletherapy and in-person groups in terms of symptoms of depression; clinical outcomes were maintained at follow-up. Although blended treatments, that is, a combination of digital components and face-to-face therapy, show promise for treating depression [160], the long-term durability of these outcomes is not yet known due to a lack of extended follow-up. Similarly, a study [186] supported the effectiveness of digital CBT tools, reporting significant decreases in the severity of clinically significant diagnoses and self-reported anxiety and depressive symptoms. However, they did not include long-term follow-up data to assess the sustainability of these improvements. A study [206] presented the protocol of a study examining the feasibility and efficiency of web-based MBCT for depression. The data collected from this study were intended to provide long-term outcomes; however, no results have been reported so far.

It is essential to underline that the efficiency of digital CBT tools may further depend on the tools being employed, the extent of support a therapist provides, and individual needs. For example, some studies, such as [161], point out that continuous support and individual interventions are necessary to establish long-term efficacy. Further, some studies, for example, [172], have focused on a short-term basis, which may not be sufficient to ensure that the treatment would be effective in the long term. Other studies [160,186] did not provide any follow-up, raising questions regarding the sustainability of the improvements. The confirmation of such benefits might further be established by ongoing research, for example, in the studies [159,206]. Taking together, the current evidence strongly suggests that digital CBT tools hold significant promise for effective long-term treatment outcomes in depression. Many studies have identified sustained improvements, equivalent efficacy to traditional therapy, and effectiveness across diverse populations. However, this is influenced by the specific tool used, the amount of support, and individual patient characteristics. Longer follow-up, especially, will be important in continuing research to consolidate these findings and explore the factors contributing to sustained benefits. Human support, personalized interventions, and continuous monitoring would appear to be key elements in maximizing the long-term effectiveness of digital CBT tools.

## 5. Discussion

The integration of technology, particularly in the form of digital tools and teletherapy, fundamentally changes the landscape of CBT for depression. Automated text messaging and other digital platforms are breaking down barriers to access, making treatment available to individuals facing geographical, mobility, or time constraints [170,200]. This means that people in remote areas or those with busy schedules can now receive the care they need. These technologies also foster better adherence to treatment plans through consistent reminders, motivational messages, and tailored feedback [203], encouraging patients to remain engaged in their therapy. Mobile apps and digital platforms further allow real-time patient progress monitoring, enabling therapists to make timely adjustments to treatment plans and potentially prevent relapses [179,181]. This shift toward digital integration represents a move toward a more accessible, engaging, personalized, and cost-effective approach to mental health care [168], actively involving patients in their treatment journey.

Despite the immense promise of integrating digital tools into CBT, specific challenges must be addressed. These include maintaining patient engagement and adherence [164], tackling accessibility and usability issues [170,203], preserving a strong therapeutic alliance in a digital environment [198], personalizing interventions to meet individual needs [157,159], ensuring patient safety and monitoring effectively [179,191], demonstrating the efficacy of these new approaches [167,189], overcoming technical obstacles, managing costs, and smoothly integrating digital tools into existing healthcare infrastructure [130]. Overcoming these challenges requires a comprehensive approach that involves creating interactive and engaging digital tools, providing sufficient technical support to users, implementing personalized and human-centered care strategies, and paying meticulous attention to safety, effectiveness, and data security. Collaboration among patients, therapists, and technology developers is essential to harness the full potential of digital tools in CBT [116]. Successful integration hinges on continuous evaluation and adaptation, always guided by a patient-centered approach.

Teletherapy-based CBT has shown comparable, and in some cases superior, efficacy to traditional in-person therapy for depression [157,192,196]. Research has consistently demonstrated significant reductions in depressive symptoms and improved quality of life across diverse patient populations [128,148,173,190]. The flexibility and accessibility of teletherapy, further enhanced by digital tools, are key to its effectiveness, allowing tailored interventions, improved engagement, and better adherence [186]. To optimize teletherapy for personalized care, it is essential to incorporate individualized treatment planning [165], real-time digital monitoring and feedback [128], flexible and supportive communication options [163,188], and advanced technologies [179,181]. The inclusion of human support alongside digital interventions, as in [116,204], has been shown to improve outcomes, and personalized feedback, as emphasized by multiple researchers [171], ensures that treatment remains responsive to the evolving needs of patients.

Digital tools facilitate the personalization of CBT using modular psychotherapies, adaptive content, and predictive algorithms designed to meet the specific needs of each patient [161,171]. This level of personalization leads to improved outcomes and greater patient satisfaction. Automation, personalized feedback, and interactive elements enhance engagement and adherence. Teletherapy and blended care models, as studied in papers [164,168], help overcome geographical and logistical barriers, making mental health services more accessible. Reducing the need for therapist contact hours, combined with stepped care and asynchronous communication, not only reduces financial costs but also increases the scalability of mental health services. Digital CBT extends the benefits of therapy to diverse and underserved populations by addressing issues such as digital literacy, inclusivity, and safety [130]. Continuous monitoring and data-driven adjustments [183] refine the therapeutic process, ensuring that interventions remain effective and responsive to patient needs.

Current research indicates that digital CBT tools offer a promising avenue for achieving long-term positive outcomes in the treatment of depression. Studies have reported sustained improvements in depressive symptoms, quality of life, and related measures over extended periods [157,170,171,182,186,193,197,200]. Digital interventions have proven effective in diverse populations, including older adults [189], individuals with Parkinson’s disease [140,141], and pregnant women [144,152,176]. Innovative approaches like the Woebot app [179], mindfulness-based CBT [184], text message-delivered CBT [172], and personalized “micro-interventions” [147] further support the potential for long-term benefits. Although efficacy may vary depending on the tool used, level of support provided, and individual patient needs, factors such as tailored interventions, human support, continuous monitoring, and individualized treatment planning contribute to optimal long-term outcomes.

**Figure 3:**
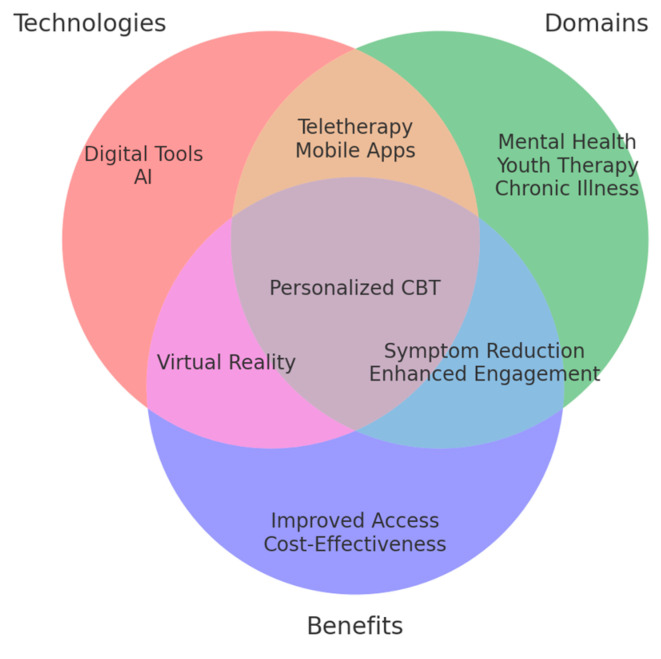
Venn diagram highlights the key overlaps between the technologies, domains, and benefits of NG-CBT.

The Venn diagram (Figure 3) highlights the intersection of technologies, domains, and benefits of NG-CBT. Central aspects, such as personalized CBT, represent the seamless integration of digital tools, application areas, and outcomes to enhance the accessibility and effectiveness of therapy.

**Figure 4:**
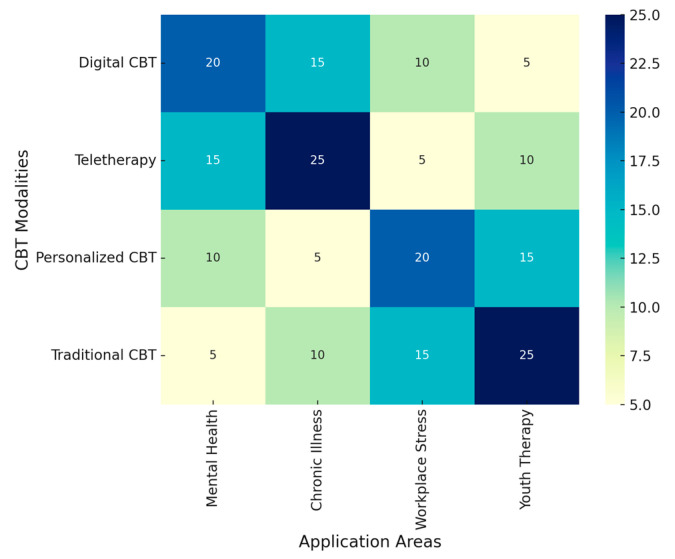
Heatmap illustrating the distribution of studies across CBT modalities and application areas.

The heatmap below (Figure 4) visualizes the relationship between the CBT modalities, for example, Digital CBT, Teletherapy, Personalized CBT, and Traditional CBT and application areas like Mental Health, Chronic Illness, Workplace Stress, and Youth Therapy. Color intensity corresponds to the relative number of studies or focuses within each combination, with darker shades indicating higher representation. For instance, the heatmap illustrates that teletherapy for chronic illness and traditional CBT for youth therapy are highly researched, while digital CBT predominates when considering mental health applications. This reflects shifts in emerging trends and gaps in the research emphasis across different therapeutic modalities and domains.

**Figure 5:**
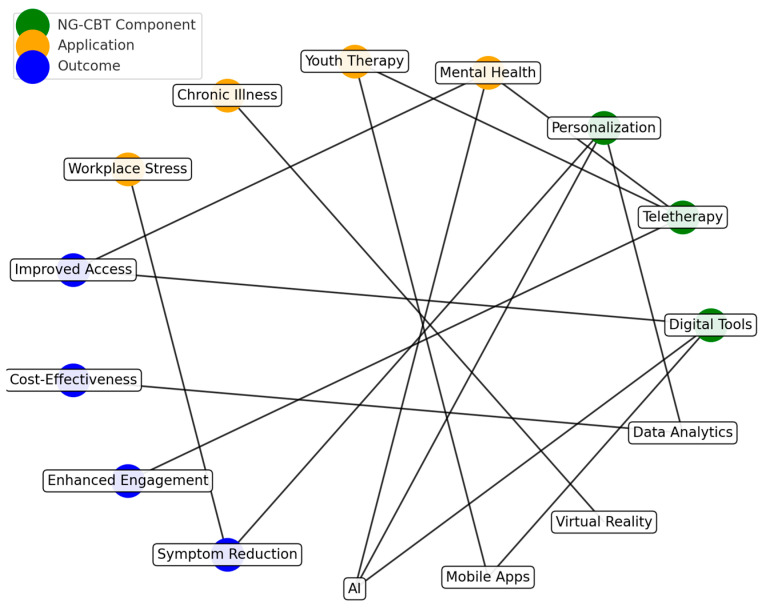
Circular Network Visualization: NG-CBT Components, Applications, and Outcomes.

The network visualization (Figure 5) illustrates the dynamic interplay between the core components of NG-CBT and the desired outcomes. Here is an analytical explanation of these connections.

1. Core Components and Their Contributions:
  ○ Digital Tools include e-therapy platforms, mobile apps, and AI-driven chatbots. Their primary role is to improve accessibility by overcoming geographical and logistical barriers, making mental health support scalable and widely available. For example, digital tools connect directly to “Improved Access”, reflecting their ability to bridge gaps in care delivery.
  ○ Teletherapy: This mode of CBT delivery uses video conferencing and remote communication technologies to maintain therapeutic engagement. Its connection to “Enhanced Engagement” highlights its strength in providing consistent and flexible care for diverse populations, including those in remote areas.
  ○ Personalization: Personalization uses patient-specific data, preferences, and conditions to tailor the interventions. Its connection to “Symptom Reduction” demonstrates the critical role of customized treatment in achieving better clinical outcomes and patient satisfaction.
2. Applications and Target Groups:
  ○ Mental Health: Central to NG-CBT, mental health applications are supported by all three components. Digital tools enable scalable solutions, teletherapy ensures consistent delivery, and personalization enhances the relevance of interventions to meet diverse patient needs.
  ○ Youth Therapy: Particularly linked to teletherapy, this application addresses the unique needs of younger populations, leveraging their familiarity with technology to improve adherence to therapy.
  ○ Chronic Illness: Personalization plays a pivotal role in addressing the complexities of depression co-occurring with chronic conditions by integrating medical and psychological data into treatment strategies.
  ○ Workplace Stress: Linked primarily to cost-effective interventions, workplace applications highlight the value of scalable digital tools and personalized strategies for managing stress at the organizational level.
3. Outcomes and System Impact:
  ○ Improved Access: Represents the impact of NG-CBT on breaking traditional barriers like location and care availability. Digital tools are critical enablers here.
  ○ Cost-Effectiveness: Reflects how NG-CBT optimizes resources through reduced therapist hours, automated monitoring, and scalable digital interventions.
  ○ Enhanced Engagement: Captures the ability of teletherapy and tailored approaches to maintain high levels of patient interaction and adherence to the therapy.
  ○ Symptom Reduction: Highlights the effectiveness of personalized and data-driven approaches in managing and alleviating depression symptoms.
4. Technological Enablers:
  ○ AI and Data Analytics: These technologies power personalization and predictive models, ensuring that interventions are dynamically adapted to patient needs.
  ○ Mobile Apps: Act as a delivery mechanism for digital CBT content, reminders, and self-monitoring tools.
  ○ Virtual Reality (VR): Adds immersive tools for specific applications like exposure therapy, especially in addressing stress or phobias.
  ○ Data Analytics: Supports cost-effective care by optimizing intervention strategies and providing insights into patient progress.

This visualization analytically demonstrates the layered and interdependent nature of the NG-CBT. These components are tightly interlinked and work to meet diverse applications and maximize outcomes through advanced technologies. This integrated framework underscores the transformative potential of NG-CBT in modern mental health care.

**Figure 6:**
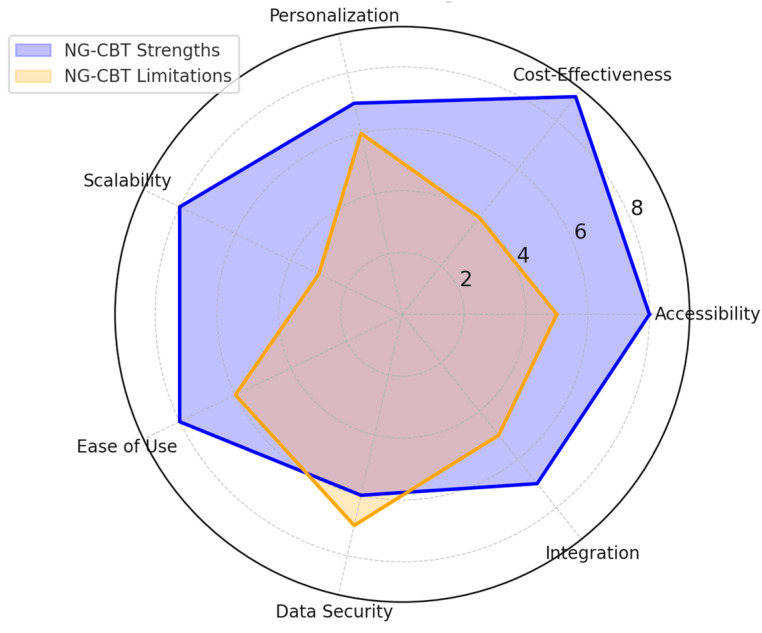
Evaluation of the strengths and challenges of NG-CBT.

Finally, the radar chart (Figure 6) visually analyzes NG-CBT’s strengths and limitations across categories such as accessibility, cost-effectiveness, personalization, scalability, ease of use, data security, and integration. It highlights the areas where NG-CBT excels, like accessibility and cost-effectiveness, while identifying challenges, particularly in data security and scalability. This chart provides a clear overview of NG-CBT’s current state, emphasizing the need for further development in key areas to maximize its impact.

### 5.1. Future Directions and Implications

The state of research on these novel delivery formats for CBT affords several directions for future research and has many implications for novel delivery and personalized impact. First, while considerable research has been conducted on each of these novel delivery formats, few studies have directly compared them to date. Given the rapid pace at which technology is evolving, efforts undertaken to evaluate these novel biotechnological methods of delivery and novel biologic agents should involve the comparison of different novel delivery formats for CBT with one another. Such research can help identify the strengths and weaknesses of these novel delivery formats and how to maximize their effects in CBT, alone or in combination.

Second, the intersection between novel CBT delivery formats and existing advantages was relatively small in this study. Novel CBT delivery formats of CBT did not appear to leverage many of the well-established benefits of CBT. Relatively few programs have aimed to maximize CBT efficacy, efficiency, or transportability. These observations suggest that ongoing work on CBT technologies could become more integrative, consistent with the ongoing convergence of CBT with other areas in psychiatry and medicine. Specifically, more significant effort is needed to align novel CBT delivery formats with the following areas: CBT innovations, CBT research methods, CBT efficacy, CBT scope of conditions, CBT portability and flexibility, CBT cost control, and CBT education and training. Integrating efforts toward this end could allow novel CBT approaches to maximize their utility.

### 5.2. Limitations

Despite the promising future of NG-CBT, several limitations must be addressed. The effectiveness of these interventions relies heavily on digital literacy, which is a hindrance to older adults and those belonging to underprivileged communities who lack experience with digital media. Additionally, data privacy and security concerns are at the forefront, as patients might not use teletherapy applications, AI-powered chatbots, and other digital platforms to their fullest because they fear data leaks and ethical complications associated with AI-powered therapy. Another downside is the restricted potential for automated interventions to mirror human therapists’ complex judgments, possibly rendering NG-CBT ineffective in complicated scenarios demanding real-time adaptation. Technological issues such as unreliable Internet connections, software glitches, and usability problems can undermine accessibility and user engagement. Whereas digital interventions provide scalability, ensuring the quality and efficacy of treatment at scale continues to be challenging, with standardized procedures and continuous therapist training being necessary. Variability in patient engagement is another problem, with some patients being very receptive to computerized CBT while others experience problems with motivation and compliance based on symptom severity or personal preference. Additionally, while NG-CBT has been successful across a range of studies, there are comparatively few comparisons with conventional face-to-face CBT, and as a result, its efficacy in the long term, especially for more complicated psychiatric conditions, cannot be ascertained. Overcoming these limitations will be important to optimize the potential of NG-CBT through the expansion of digital literacy training, improving data security, improving AI-driven personalization, and providing comprehensive technical support to foster effective and equitable mental health care.

## 6. Conclusions

This review systematically explored the available evidence concerning the application of digital and teletherapy for depression, both as stand-alone and integrated modalities for people with depression. The review highlights the feasibility and acceptability of digital and teletherapy formats across a variety of therapy techniques and modalities for treating people with depression. Our review also identified a range of individual and contextual factors that helped guide the development of an integrated approach for digital teletherapy targeting depression. These factors contribute to personalized mental treatment and an integrated approach, including personal choice and flexibility, the range of individual abilities and skills, patterns of social health, and individual decisions on whether a stand-alone or combined approach of digital teletherapy delivery format would best fit their abilities, accessibility to automated and interactive services, personal education, and therapeutic functions of teletherapy, access to guided or self-guided services, privacy and confidentiality, and engaging factors.

In conclusion, most people with depression continue to seek and benefit from mental health interventions delivered by traditional providers. They feel comfortable expressing their feelings to providers and receiving consistent guidance regarding their depression symptoms. New and innovative CBT and digital teletherapy services that provide clinically guided care are not intended to replace traditional healthcare professional services. Instead, technology serves to complement conventional mental health delivery services for depression treatment, providing support and leveraging the skills of individuals who might not have access to or are hesitant to seek in-person mental health services to improve depressive symptoms. These findings suggest offering choices from various modalities, from digital to in-person, and integrating site-specific technology, providing broad advantages to mitigate mental health disparities related to sociodemographics, increase public health awareness, improve clinical capacities, and occupy pay-for-performance models. Associations should consider these findings in patient care practices and devise strategies to promote increased use for treating depressive disorders.

## Figures and Tables

**Table 1:** Research articles of systematic analysis (n = 81).

Authors	Study Objectives	Main Findings	Participant Count	Intervention	**Hypotheses**
Aguilera et al., (2017) [127]	Automated Text Messaging as an Adjunct to CBT for Depression: A Clinical Trial	Automated text messaging as an adjunct to group CBT for depression increased therapy engagement but did not improve depression outcomes.	Total: 85 -Control condition: 40 -Text messaging adjunct: 45	-Daily mood monitoring messages -Daily messages reiterating the weekly CBT content -Medication and appointment reminders The therapist also used the mood data and responses from the text messages during the group CBT sessions.	-The text messaging adjunct will increase the number of group therapy sessions attended -The text messaging adjunct will increase the duration of therapy attended -The text messaging adjunct will lead to greater reductions in depressive symptoms (PHQ-9) compared to the control condition.
Alavi et al., (2023) [128]	Investigating the effectiveness of incorporating a stepped care approach into electronically delivered CBT for depression	A stepped-care model of electronically delivered CBT for depression can be an effective and scalable approach to providing personalized care.	Total: 79 -e-CBT group: 53 -e-CBT with stepped care group: 26	-12–13 weeks of e-CBT for all participants -Additional “stepped care” interventions for the experimental group (n = 26), based on their individual needs as assessed by questionnaire scores and other data	-To examine the efficacy of a stepped-care e-CBT model for depression through a reduction in depressive symptoms. -To develop a decision-making process that can effectively allocate the appropriate level of care for each patient.
Anguera et al., (2017) [129]	Improving late-life depression and cognitive control through the use of therapeutic video game technology: A proof-of-concept randomized trial	Therapeutic video game technology can enhance late-life depression and cognitive control.	Total: 22	-Project: EVO™, a mobile iPad intervention based on the NeuroRacer video game, where participants played the game for 20 min, 5 days per week, for 4 weeks. -Problem-Solving Therapy (PST), an 8-week intervention with 3 phases: 3 weeks of psychoeducation, 4 weeks of independent practice, and 2 weeks of relapse prevention.	-To explore the impact of a cognitive control intervention (Project: EVO) on cognitive control network, mood, and disability in people with late-life depression and compare it to the impact of problem-solving therapy (PST). -To explore the association between cognitive control network activation (as measured by behavioral assessments) and mood and functional disability. -To determine the acceptability of the cognitive control intervention (Project: EVO) for depressed individuals.
Atik et al., (2022) [130]	Patient and Therapist Expectations for a Blended CBT Program for Depression: Qualitative Exploratory Study	Patients and therapists have positive attitudes toward blended CBT for depression, with expectations for customizable digital tools to track mood and facilitate therapy.	Total: 28	-CBT participants: Qualitatively assess how patients and therapists perceive these tools, focusing on their expectations and preferences rather than implementing a specific intervention	-Digital tools would support therapy by offering features such as mood tracking, homework facilitation, and personalized feedback. -Therapists were expected to view these tools as complementary to face-to-face therapy, helping to maintain a strong therapeutic alliance while reducing workloads. -Both patients and therapists likely prioritized customization and usability, expecting tools to adapt to individual therapy needs and goals.
Berking et al., (2013) [131]	Emotion Regulation Skills Training Enhances the Efficacy of Inpatient CBT for Major Depressive Disorder: A RCT	Integrating emotion regulation skills training enhances the efficacy of inpatient CBT for major depressive disorder.	Total: 432	-CBT participants received at least one 45-min session of individual therapy and four 45-min sessions of group psychotherapy per week specifically targeting depression, as well as four 45-min sessions of transdiagnostic group therapy based on problem-solving therapy. -Emotion regulation training (ERT)—participants in the CBT-ERT condition received an abbreviated version of the Affect Regulation Training (ART) program, consisting of four 1.5-h sessions and two 45 min sessions. -Supplemental treatments—participants also received disorder-specific group therapy for any comorbid disorders, as well as physiotherapy and medical treatment as needed.	-Supplementing routine CBT with a systematic ERT (CBT-ERT) would enhance the reduction of depressive symptoms and increase response and remission rates among participants. -CBT-ERT would be superior to routine CBT for improving well-being, negative affect, and ER skills, with specific effects on certain ER skills, and the effects would not be moderated by pre-treatment factors like ER skills, sex, age, or type of depression diagnosis.
Bronswijk et al., (2019) [132]	Precision medicine for long-term depression outcomes using the Personalized Advantage Index approach: cognitive therapy (CT) or interpersonal psychotherapy?	The Personalized Advantage Index can predict the optimal psychotherapy (cognitive therapy or interpersonal psychotherapy) for long-term depression outcomes.	Total: 151 -CT: 76 -Interpersonal Psychotherapy (IPT): 75	-CT, received by 76 participants -Interpersonal psychotherapy (IPT), received by 75 participants The abstract does not provide any information about the frequency, duration, or amount/dose of the interventions.	-The Personalized Advantage Index (PAI) approach can be used to predict optimal treatment for long-term depression outcomes in addition to acute-phase psychotherapy. -Individuals assigned to their PAI-indicated treatment will have better long-term depression outcomes compared to those assigned to their PAI-non-indicated treatment. -Long-term PAI predictions will be different from predictions for acute benefit.
Brothers et al., (2011) [133]	Cancer patients with major depressive disorder: testing a biobehavioral/CBT	A combined biobehavioral intervention and CBT was effective in reducing depressive symptoms in cancer patients.	Total: 36	-The intervention was a combined biobehavioral intervention (BBI) and CBT intervention for cancer patients with major depressive disorder (MDD). It consisted of 12–20 individual, 75 min sessions, with the initial 12 sessions being weekly.	-The combined BBI/CBT intervention would be effective in reducing depressive symptoms in cancer patients with major depressive disorder. -Individual differences such as cancer-related stress, history of prior depressive episodes, and presence of comorbid anxiety disorders would be associated with depressive symptom severity. -The BBI/CBT intervention would improve secondary outcomes such as quality of life, pain, and fatigue in cancer patients.
Brown et al., (2021) [134]	Reinforcement Learning Disruptions in Individuals With Depression and Sensitivity to Symptom Change Following CBT	Reinforcement learning disruptions are associated with depression symptoms, and symptom improvement following CBT is linked to the normalization of learning parameters.	Total: 101	-The intervention was a 12-week course of standard, manual-guided CBT provided to 28 out of the 69 participants with depression.	-Distinct processes in reward and loss learning, as captured by computational model-derived parameters, would be associated with symptoms of anhedonia and negative affect, respectively. -Changes in these reward and loss learning parameters would be correlated with symptom improvement after CBT treatment.
Calleo et al., (2015) [135]	A Pilot Study of a CBT for Anxiety and Depression in Patients With Parkinson’s Disease	This pilot study found that a CBT program delivered via telephone or in-person is feasible for treating anxiety and depression in Parkinson’s disease patients.	Total: 16	-The intervention was a CBT program that included tools for anxiety, depression, and healthy living with Parkinson’s disease symptoms. -The sessions were delivered either by telephone or in-person, based on patient preference, with 67% of sessions conducted by telephone. 80% of participants completed the full CBT treatment.	-The CBT intervention will be more effective than enhanced usual care in reducing anxiety and depression in patients with Parkinson’s disease. -The CBT intervention, including the option for telephone-based sessions, will be a feasible treatment approach for patients with Parkinson’s disease.
Carter et al., (2013) [136]	Psychotherapy for depression: a randomized clinical trial comparing schema therapy and CBT	Schema therapy and CBT are comparable in efficacy for treating depression.	Total: 100	-CBT -Schema Therapy (ST) -Participants received the assigned therapy weekly for the first 6 months, followed by monthly sessions for the next 6 months.	-CBT and ST have comparable efficacy in the treatment of depression -There are no differential treatment effects between CBT and ST for individuals with chronic depression or comorbid personality disorders.
Cheng et al., (2020) [137]	Depression prevention in digital CBT for insomnia: Is rumination a mediator?	Digital CBT for insomnia reduces depression by decreasing rumination.	Total: 658 -dCBT-I: 358 -Control: 300	-The intervention was digital CBT for Insomnia (dCBT-I).	-Reductions in rumination (PTQ) were significantly larger in the dCBT-I condition compared to the control. -Reductions in rumination significantly mediated the improvement in post-treatment insomnia severity and post-treatment depression severity associated with the dCBT-I condition. -Reductions in rumination also significantly mediated the prevention of clinically significant depression via dCBT-I.
Clarke et al., (2016) [138]	CBT in Primary Care for Youth Declining Antidepressants: A Randomized Trial	Brief CBT is a viable alternative to antidepressants for treating depression in adolescents in primary care.	Total: 212 -TAU control condition: 106 -TAU plus CBT condition: 106	-The intervention was brief CBT delivered in a primary care setting. -The CBT consisted of two 4-session modules—one on CT to address unrealistic thinking and one on behavioral activation to increase pleasant activities. -Participants could stop after the first module if they were nearly recovered or continue to the second module if they were partial or non-responders. Up to 6 additional continuation contacts were also permitted. -The CBT was delivered by therapists with at least a master’s degree and experience delivering CBT, with biweekly supervision.	-Brief CBT delivered in primary care is a viable alternative treatment for depressed adolescents who have declined or discontinued antidepressant medication. -Brief CBT can impart clinical benefits beyond usual care for depressed adolescents who are unreceptive to pharmacotherapy.
Coyne et al., (2022) [139]	Replicating patient-level moderators of CBT and IPT’s comparative efficacy for depression	The paper examines patient characteristics that moderate the comparative efficacy of CBT and interpersonal psychotherapy for depression.	Total: 80 -CBT: 41 -IPT: 39	-CBT for 16 weeks -Interpersonal Psychotherapy (IPT) for 16 weeks	-Replicating significant ATIs that were previously established in a single study. -Replicating significant ATIs that were previously examined twice, with only one study demonstrating a moderating effect.
Dobkin et al., (2018) [140]	Personalized Telemedicine for Depression in Parkinson’s Disease: A Pilot Trial	A personalized cognitive-behavioral telemedicine program for depression in Parkinson’s disease showed improvements in various outcomes.	Total: 34	-A 10-module self-help workbook tailored for depression in Parkinson’s disease, which could be used either as a stand-alone intervention with minimal therapist support or as a supplement to formal therapy -Formal telephone-administered CBT sessions The study looked at both the self-help workbook and the formal therapy sessions as part of the intervention and found improvements in depression, anxiety, quality of life, sleep, negative thoughts, and caregiver burden, regardless of which modality was used over the course of the 4-month study.	-The feasibility and impact of a personalized cognitive-behavioral telemedicine program for depression in Parkinson’s disease patients -Improvements in depression, anxiety, quality of life, sleep, negative thoughts, and caregiver burden, independent of treatment modality (guided self-help vs formal telephone-based psychotherapy).
Dobkin et al., (2020) [141]	Telephone-based CBT for depression in Parkinson’s disease	Telephone-based CBT is an effective intervention for depression in Parkinson’s disease patients.	Total: 72	-The intervention was telephone-based cognitive-behavioral treatment (T-CBT) provided in 10 sessions, weekly for the first 3 months and then monthly for the 6-month follow-up period. -The CBT targeted negative thoughts and behaviors and also involved training care partners to help the participants practice healthy habits.	-T-CBT would alleviate depressive symptoms significantly more than treatment as usual (TAU) in patients with Parkinson’s disease and depression.
Donker et al., (2013) [142]	Predictors and moderators of response to Internet-delivered Interpersonal Psychotherapy and CBT for depression	Age moderates the effectiveness of Internet-delivered IPT versus CBT for depression, with younger people benefiting more from IPT and older people from CBT.	Total: 1843 -Interpersonal Psychotherapy (IPT): 620 -CBT: 610 -MoodGYM (control): 613	-Interpersonal Psychotherapy (IPT) -CBT -The interventions were delivered over a 4-week period.	-Identifying predictors and moderators of response to Internet-delivered Interpersonal Psychotherapy (IPT) and CBT for depression -Comparing the effectiveness of Internet-delivered IPT and CBT to an active control intervention (MoodGYM) -Examining whether certain socio-demographic, clinical, and psychological characteristics moderate the effectiveness of the interventions.
Dwight-Johnson et al., (2011) [143]	T-CBT for Latino patients living in rural areas: a randomized pilot study	T-CBT improved depression outcomes in rural Latino patients.	Total: 101	-The intervention was culturally tailored, telephone-based CBT delivered in 8 sessions by trained bilingual therapists. -The CBT focused on behavioral activation and cognitive restructuring techniques, and the initial session also included an assessment of clinical history and motivation. -Therapists could also make brief supportive phone calls between sessions if needed.	-Culturally tailored, telephone-based CBT would be effective in improving depression outcomes among Latino primary care patients in rural settings. -The telephone-based CBT intervention would lead to greater improvements in depression outcomes and higher patient satisfaction compared to usual primary care.
Felder et al., (2021) [144]	RCT of digital cognitive behavior therapy (dCBT) for prenatal insomnia symptoms: Effects on postpartum insomnia and mental health	dCBT for insomnia during pregnancy leads to benefits for postpartum insomnia, depression, and anxiety.	Total: 208	-The intervention was 6 weekly sessions of dCBT-I delivered through the Sleepio program. -The dCBT-I intervention included components like sleep restriction, stimulus control, cognitive therapy, relaxation techniques, and sleep hygiene education.	-Participants randomized to dCBT-I during pregnancy would have significantly greater improvements in insomnia symptoms from baseline to 3 and 6 months postpartum relative to participants randomized to standard care. -Participants randomized to dCBT-I during pregnancy would have significantly greater improvements in depressive and anxiety symptoms from baseline to 3 and 6 months postpartum relative to participants randomized to standard care. -The study explored condition differences in rates of probable major depression in the full sample and among the subset of participants with minimal depressive symptoms at baseline, as well as in rates of moderate-to-severe anxiety in the full sample and among the subset of participants with minimal to mild anxiety symptoms at baseline.
Fitzpatrick et al., (2017) [145]	Delivering CBT to Young Adults With Symptoms of Depression and Anxiety Using a Fully Automated Conversational Agent (Woebot, Ver 1.0.1): A RCT	A fully automated conversational agent can effectively deliver CBT for reducing symptoms of depression in young adults.	Total: 70	-The intervention was a fully automated conversational agent called “Woebot” that delivered CBT content in a conversational format over a 2-week period, with participants engaging with the agent an average of 12 times during that period.	-Conversation with a therapeutic process-oriented conversational agent (Woebot) would lead to greater improvement in symptoms of depression and anxiety relative to an information control group. -Receiving psychoeducational material in a conversational manner would be more acceptable to those who received it (the Woebot group) compared to the information control group.
Forsell et al., (2017) [146]	Internet-delivered CBT for antenatal depression: A randomized controlled trial	Internet-delivered CBT is effective for treating antenatal depression.	Total: 42 -ICBT: 21 -TAU: 21	-The intervention was a 10-week Internet-delivered CBT (ICBT) program for antenatal depression, provided as an add-on to the participants’ usual antenatal care.	-The efficacy of a pregnancy-adapted ICBT program for antenatal depression -The acceptability and adherence to the ICBT program for antenatal depression.
Frank et al., (2022) [147]	Personalized digital intervention for depression based on social rhythm principles adds significantly to outpatient treatment	A personalized digital intervention for depression based on social rhythm regulation principles significantly improves outcomes when added to outpatient treatment.	Total: 133	-Continuous monitoring of depression-relevant behavior patterns using smartphone sensors -Provision of personalized “micro-interventions” based on the patient’s behavioral data, delivered via push notifications to the patient’s phone approximately every 2–3 days over 16 weeks -Psychoeducational “Learning Modules” provided to patients over the first 3 weeks of the intervention, which explained the rationale for regular routines and how they can help reduce depression symptoms	-The Cue digital intervention platform would lead to greater improvement in depressive symptoms compared to a control condition of just smartphone-based behavioral monitoring. -The Cue intervention would also show benefits in the subgroup of participants who were experiencing moderately severe to severe depression at the start of the study.
Gilliam et al., (2019) [148]	A Trial of Sertraline or CBT for Depression in Epilepsy	This paper evaluates the comparative effectiveness of sertraline and CBT for depression in people with epilepsy.	Total: 140	-Sertraline, starting at 50 mg per day and increasing by 50 mg every 2 weeks as needed, up to a maximum of 200 mg per day. -CBT, consisting of weekly 1-h sessions with a licensed therapist using a standardized, manual-based approach.	-To compare the effectiveness of sertraline and CBT in treating depression, improving quality of life, and affecting seizures and adverse effects in people with epilepsy. -To test the hypothesis that sertraline is associated with a greater than 15% occurrence of generalized tonic-clonic (GTC) seizures compared to CBT.
Giosan et al., (2014) [149]	Evolutionary cognitive therapy versus standard cognitive therapy for depression: a protocol for a blinded, randomized, superiority clinical trial	This study compares the efficacy of evolutionary-driven cognitive therapy versus standard cognitive therapy for treating depression.	Total: 12	-Evolutionary-driven cognitive therapy (ED-CT) -Cognitive therapy (CT) Both interventions involve 12 psychotherapy sessions.	-The evolutionary-driven cognitive therapy (ED-CT) protocol will be more effective than the standard cognitive therapy (CT) protocol in reducing depressive symptoms and the proportion of participants meeting the criteria for a depression diagnosis after treatment. -Integrating insights from evolutionary theories of depression into a cognitive therapy protocol will result in improved treatment outcomes compared to standard cognitive therapy.
Graaf et al., (2010) [150]	Predicting outcome in computerized CBT for depression in primary care: A randomized trial	Certain patient characteristics predict better outcomes for computerized CBT, usual care, or their combination for depression.	Total: 303 -Unsupported online CCBT: ~101 -Treatment as usual (TAU): ~101 -CCBT and TAU combined (CCBT&TAU): ~101	-Unsupported online computerized CBT (CCBT) -Treatment as usual (TAU) -CCBT and TAU combined (CCBT&TAU)	-Certain pre-treatment and short-term improvement variables would moderate the 12-month outcomes of CCBT, usual care, and CCBT combined with usual care for depression. -Patients with higher levels of positive responding would have better outcomes in CCBT compared to usual care. -Patients with a parental psychiatric history or major depressive disorder diagnosis would have better outcomes in CCBT combined with usual care compared to usual care. -Certain patient characteristics (current employment, low pre-treatment illness severity, short-term improvement) would predict better outcomes, regardless of the treatment condition.
Haller et al., (2016) [151]	Integrated CBT Versus Cognitive Processing Therapy for Adults With Depression, Substance Use Disorder, and Trauma	Integrated CBT and cognitive processing therapy are both effective for treating depression, substance use disorder, and trauma in veterans.	Total: 123	-12 weeks of group-ICBT, twice per week -12 individual follow-up sessions using either ICBT or modified cognitive processing therapy (CPT-M) that integrated substance use disorder treatment	-Adding a trauma-focused treatment (CPT-M) after an initial group-based ICBT would improve treatment outcomes for participants with SUD, depression, and PTSD compared to those who received only the initial ICBT without the additional trauma-focused treatment. -The trauma-focused CPT-M treatment in Phase 2 would lead to better outcomes compared to the non-trauma-focused ICBT treatment in Phase 2, particularly for participants with PTSD.
Henry et al., (2020) [152]	Insomnia as a mediating therapeutic target for depressive symptoms: A sub-analysis of participant data from two large RCTs of a digital sleep intervention	Digital CBT for insomnia can improve both insomnia and depressive symptoms, with insomnia improvement mediating the effects of depression.	Total: 3352	-The intervention was a fully automated digital CBT intervention for insomnia called Sleepio.	-Digital CBT for insomnia will improve insomnia and depressive symptoms in participants with clinically significant insomnia and depression. -Improvements in insomnia symptoms will mediate the effects of the digital CBT intervention on depressive symptoms. -There will be no moderators of the effectiveness of the digital CBT intervention on insomnia and depressive symptoms.
Ho et al., (2020) [153]	The effect of self-help CBT for insomnia on depressive symptoms: An updated meta-analysis of RCTs	Self-help CBT for insomnia is effective in reducing depressive symptoms.	Total: 5945	-Self-help CBT for insomnia (self-help CBT-I).	-Self-help CBT for insomnia (CBT-I) is effective in reducing depressive symptoms. -Self-help CBT-I is effective in reducing insomnia symptoms.
Hsia et al., (2022) [154]	Randomized, Controlled Trial of a Digital Behavioral Therapeutic Application to Improve Glycemic Control in Adults With Type 2 Diabetes	A digital CBT app improved glycemic control in adults with type 2 diabetes.	Total: 669	-The intervention was a digital therapeutic application (app) delivering CBT designed to improve glycemic control in patients with type 2 diabetes.	-The primary hypothesis tested in this study was that a digital therapeutic application delivering CBT would improve glycemic control (as measured by HbA1c) in patients with type 2 diabetes compared to a control app.
Huibers et al., (2015) [155]	Predicting Optimal Outcomes in Cognitive Therapy (CT) or Interpersonal Psychotherapy for Depressed Individuals Using the Personalized Advantage Index Approach	Predicting optimal outcomes in CT or interpersonal psychotherapy for depression using the Personalized Advantage Index approach.	Total: 134	-CT: 12 to 20 individual sessions of 45 min each, based on the manual by Beck et al. -Interpersonal psychotherapy (IPT): 12 to 20 individual sessions of 45 min each. -The interventions were delivered by 10 licensed therapists (5 in each condition) with an average of 9.1 years of clinical experience.	-The Personalized Advantage Index (PAI) approach developed by DeRubeis et al. can be replicated when comparing two psychotherapies (cognitive therapy and interpersonal therapy) that have been shown to have equivalent overall outcomes. -The study will be able to identify predictors and moderators of treatment outcomes for cognitive therapy and interpersonal therapy, and these can be used to generate PAI values to predict the optimal treatment for individual patients. -Patients who receive the psychotherapy predicted to be optimal for them based on the PAI will have better outcomes than those who receive the non-optimal psychotherapy.
Interian et al., (2022) [156]	A Pilot Study of Telehealth Mindfulness-Based Cognitive Therapy (MBCT) for Depression in Parkinson’s Disease	A pilot study found that telehealth mindfulness-based cognitive therapy for depression in Parkinson’s disease was feasible and beneficial.	Total: 15	-The intervention was a telehealth MBCT program adapted for Parkinson’s disease (MBCT-PD), which participants completed in 9 sessions.	-The telehealth MBCT-PD intervention will be feasible and beneficial for individuals with Parkinson’s disease and clinically significant depression. -The telehealth MBCT-PD intervention will lead to improvements in depression, anxiety, and quality of life in individuals with Parkinson’s disease and clinically significant depression.
Johansson et al., (2012) [157]	Tailored vs. Standardized Internet-Based CBT for Depression and Comorbid Symptoms: A RCT	Tailored Internet-based CBT is more effective than standardized treatment for depression with comorbid symptoms.	Total: 121	-Standardized Internet-based CBT (ICBT) consisting of 8 self-help chapters on behavioral activation, cognitive restructuring, sleep management, general health advice, and relapse prevention, lasting 10 weeks with e-mail support from a therapist. -Tailored ICBT consisting of 25 possible chapters on depression, panic, social anxiety, worry, stress management, concentration problems, problem-solving, mindfulness, and relaxation, with an average of 9.7 chapters assigned to each participant based on their individual needs, also lasting 10 weeks with e-mail support from a therapist.	-Both the tailored ICBT treatment and the standardized ICBT treatment will be effective, with the tailored treatment showing a larger effect. -The online discussion group will have a smaller effect than the two ICBT treatment conditions. -The tailored ICBT treatment will be more effective than the standardized ICBT treatment for participants with higher initial depression severity.
Kalapatapu et al., (2014) [158]	CBT in Depressed Primary Care Patients with Co-Occurring Problematic Alcohol Use: Effect of Telephone-Administered vs. Face-to-Face Treatment—A Secondary Analysis	Telephone-administered and face-to-face CBT have similar treatment adherence and efficacy for depression in primary care patients with co-occurring problematic alcohol use.	Total: 103 -Face-to-face CBT: 53 -T-CBT: 50	-The intervention was 18 sessions of CBT, delivered either face-to-face or via telephone, with each session lasting 45 min. -The sessions were structured as two sessions per week for the first 2 weeks, followed by 12 weekly sessions, and then two final booster sessions in the last 4 weeks. -All participants also received a workbook with CBT content.	-T-CBT participants would attend significantly more sessions than those receiving face-to-face CBT -Significantly fewer participants would discontinue T-CBT before session 18 compared with face-to-face CBT -T-CBT participants would not have inferior depression outcomes compared to face-to-face CBT participants.
Kayser et al., (2022) [159]	Layperson-Facilitated Internet-Delivered CBT for Homebound Older Adults With Depression: Protocol for an RCT	This protocol describes a pilot RCT to assess the efficacy of a layperson-facilitated internet-delivered CBT program for homebound older adults with depression.	Total: 70 -Active treatment: 35 -Waitlist control: 35	-The intervention is a 9-session internet-delivered CBT (iCBT) program called “Empower@Home” that is tailored for homebound older adults with depression. -The program consists of 9 web-based sessions delivered over 10 weeks, with each session including short videos, interactive exercises, and a workbook. -The sessions are supplemented by weekly check-in calls with trained layperson “Empower Coaches” who provide support and guidance.	-The Empower@Home intervention will be feasible and acceptable for low-income, homebound older adults with depression. -The Empower@Home intervention will reduce depressive symptoms in low-income, homebound older adults. -The Empower@Home intervention will improve psychosocial functioning and health-related quality of life in low-income, homebound older adults.
Kemmeren et al., (2019) [160]	Unraveling the Black Box: Exploring Usage Patterns of a Blended Treatment for Depression in a Multicenter Study	Blended CBT for depression shows variability in usage patterns between primary and specialized care settings.	Total: 1143	-Face-to-face (FTF) therapy sessions with a therapist, with a recommended frequency of 6–10 sessions over 7–20 weeks. -Web-based modules delivered through the Moodbuster platform, including two mandatory modules (Introduction and Psychoeducation) and four optional modules (Behavioral Activation, Cognitive Restructuring, Problem Solving, and Physical Exercise). Patients were expected to complete at least the Cognitive Restructuring and Behavioral Activation modules. -Asynchronous web-based feedback from the therapist to the patient between FTF sessions. -A mobile app for daily mood monitoring, with patients prompted to rate their mood once per day.	-Describe the usage of the different components of a blended CBT (bCBT) for depression -Reflect on the actual usage of the blended treatment compared to the intended application in each country -Compare usage patterns of bCBT between primary and specialized care settings -Identify who complies with a blended treatment approach based on usage intensity and integration of face-to-face and web-based components.
Kim et al., (2014) [161]	Computer-assisted CBT for pregnant women with major depressive disorder	Computer-assisted CBT shows promise as a treatment for major depressive disorder in pregnant women.	Total: 12	The intervention was computer-assisted CBT (CCBT) consisting of 8 sessions over 6–8 weeks, with 3.75 total hours of direct therapist contact. Each session involved an initial 25–50 min session with a therapist, followed by a 25–35 min session with the computer software program “Good Days Ahead”, which provided multimedia content to teach the basic principles of CBT.	-CCBT would be an acceptable treatment for pregnant women with major depressive disorder (MDD) -CCBT would significantly decrease depressive symptoms in pregnant women with MDD.
Kobak et al., (2015) [162]	Integrating technology into CBT for adolescent depression: a pilot study	A pilot study found that integrating technology (online training, in-session tablets, text messaging) into CBT for adolescent depression is feasible and may improve therapeutic alliance.	Total: 72	-Online therapist training on CBT for adolescent depression -In-session use of tablets to teach CBT concepts and skills to patients -Text messaging for between-session homework reminders and self-monitoring	-The feasibility and effectiveness of integrating technology (online training, tablets, and text messaging) into CBT for treating adolescent depression. -The effectiveness of the technology-enhanced CBT intervention as measured by changes in clinician knowledge, user satisfaction, depression symptoms, and therapeutic alliance.
Koenig et al., (2016) [163]	Religiously-Integrated CBT for Major Depression in Chronic Medical Illness: Review of Results from a Randomized Clinical Trial	Religiously-integrated CBT is as effective as standard CBT for treating major depression in patients with chronic medical illness.	Total: 132 -CCBT: 67 -RCBT: 65	-Religiously-integrated CBT (RCBT) -Conventional CBT (CCBT) -Both interventions consisted of 10 sessions of 50 min each, delivered over 12 weeks, and conducted remotely, largely by telephone.	-RCBT is as effective as CCBT in the treatment of major depressive disorder (MDD) in patients with chronic medical illness. -The effectiveness of RCBT and CCBT may be moderated by genetic factors, specifically polymorphisms in genes related to serotonin and monoamine neurotransmission.
Kooistra et al., (2014) [164]	Blended vs. face-to-face cognitive-behavioral treatment for major depression in specialized mental health care: study protocol of a randomized controlled cost-effectiveness trial	This study protocol evaluates the feasibility, acceptability, and cost-effectiveness of blended CBT compared to traditional face-to-face therapy for major depression.	Total: 150	-Blended CBT: 10 face-to-face sessions and nine online sessions, provided alternately on a weekly basis. -Traditional CBT: 20 weekly face-to-face sessions.	-Blended CBT, which combines online and face-to-face sessions, is more cost-effective than traditional face-to-face CBT while maintaining similar clinical outcomes. -Blended CBT is non-inferior or superior to traditional face-to-face CBT in terms of clinical outcomes for patients with major depressive disorder.
Lichstein et al., (2013) [165]	Telehealth CBT for co-occurring insomnia and depression symptoms in older adults	Telehealth CBT can effectively treat comorbid insomnia and depression in older adults.	Total: 5	-The intervention was 10 weekly 50 min sessions of CBT for insomnia and depression delivered via Skype telehealth. The first five sessions were evenly split between insomnia and depression treatment, while the last five sessions focused more on depression. -The insomnia treatment components included sleep hygiene, sleep compression, stimulus control, and relaxation, while the depression treatment included cognitive-behavioral techniques.	-Telehealth-delivered CBT is feasible for treating comorbid insomnia and depression in older adults living in rural areas. -Telehealth-delivered CBT is effective for improving insomnia and depression symptoms in older adults living in rural areas. -Older adults, including those with limited technological experience, will tolerate and accept receiving CBT via telehealth.
Ludman et al., (2007) [166]	A randomized trial of telephone psychotherapy and pharmacotherapy for depression: continuation and durability of effects	Telephone-based CBT plus care management improves depression outcomes compared to usual care.	Total: 393	-The intervention was telephone-based CBT plus care management provided to primary care patients beginning antidepressant treatment. -The intervention was provided over a period of at least 6–18 months.	-Telephone-based CBT plus care management would lead to better long-term outcomes compared to usual care for primary care patients beginning antidepressant treatment. -The phone therapy group would have significantly lower depression scores compared to the usual care group from 6 to 18 months.
Lutz et al., (2021) [167]	Prospective evaluation of a clinical decision support system in psychological therapy	A prospective study evaluating a digital decision support system for cognitive-behavioral therapy, finding it can improve outcomes when therapists follow the recommended treatment strategies.	Total: 538 -Intervention group: 335 Treatment-as-usual (control) group: 203	-Clinical strategy recommendations for the first 10 sessions, including problem-solving, motivation-oriented, or a mix of both strategies. -Adaptive recommendations for patients at risk of treatment failure, including psychometric feedback enhanced with clinical problem-solving tools.	-The effect of providing therapists with specific treatment strategy recommendations (problem-solving, motivation-oriented, or a mix) for the first 10 sessions on treatment outcomes. -The effect of providing therapists with psychometric feedback enhanced with clinical problem-solving tools on treatment outcomes.
Ly et al., (2015) [168]	Smartphone-Supported versus Full Behavioural Activation for Depression: A Randomised Controlled Trial	A blended smartphone-supported behavioral activation treatment for depression was not found to be non-inferior to a full face-to-face behavioral activation treatment but reduced therapist time.	Total: 93 -Blended treatment: 46 -Full BA treatment: 47	-Blended treatment: 4 face-to-face sessions over 9 weeks, plus a smartphone app used between sessions -Full behavioral activation (BA) treatment: 10 face-to-face sessions over 10 weeks without a smartphone app	-The blended treatment with four face-to-face sessions plus a smartphone app would be non-inferior to the standard 10-session full behavioral activation (BA) treatment. -The blended treatment would reduce therapist time compared to the full BA treatment while maintaining the same treatment quality.
Manber et al., (2008) [169]	CBT for insomnia enhances depression outcomes in patients with comorbid major depressive disorder and insomnia	CBT for insomnia enhances depression outcomes in patients with comorbid major depressive disorder and insomnia.	Total: 30	-Escitalopram (EsCIT) antidepressant medication -7 individual therapy sessions of CBT for insomnia (CBT-I) -7 individual therapy sessions of a control therapy called “quasi-desensitization”	-Adding CBT for insomnia (CBT-I) to the antidepressant medication escitalopram (EsCIT) will be more effective in treating depression and insomnia in individuals with comorbid major depressive disorder (MDD) and insomnia, compared to EsCIT plus a control intervention. -EsCIT + CBT-I will lead to higher rates of remission from both depression and insomnia, as well as greater improvements in sleep measures, compared to EsCIT plus a control intervention.
Månsson et al., (2013) [170]	Development and Initial Evaluation of an Internet-Based Support System for Face-to-Face CBT: A Proof of Concept Study	An Internet-based support system can effectively blend with face-to-face CBT, facilitating communication and reducing therapist drift.	Total: 23	-The intervention was a blended treatment format that combined 8–9 weeks of face-to-face CBT with an Internet-based support system that provided access to CBT components like psychoeducation, homework assignments, and communication between sessions. The therapists controlled the content that patients could access on the platform.	-The Internet-based support system would contribute positively to patient adherence to the face-to-face CBT treatment. -The clinical outcomes (anxiety, depression, quality of life) achieved with the blended face-to-face CBT and Internet-based support system would be similar to those achieved with face-to-face CBT alone. -The users (both patients and therapists) would perceive the Internet-based support system as beneficial and would use it in positive ways.
Mantani et al., (2017) [171]	Smartphone CBT as an Adjunct to Pharmacotherapy for Refractory Depression: RCT	Smartphone-based CBT is effective as an adjunct to pharmacotherapy for treatment-resistant depression.	Total: 164	-Switching antidepressant medication to either escitalopram (5–10 mg/day) or sertraline (25–100 mg/day), with the previous antidepressant tapered off by week 5. -Use of a smartphone-based CBT app called “Kokoro-app”, which consisted of 8 sessions over approximately 8 weeks, with each session taking around 20 min to complete.	-Adding a smartphone-based CBT app to medication change is more effective than medication change alone in reducing depression severity among patients with antidepressant-resistant major depression. -The smartphone-based CBT app is effective in improving depression outcomes compared to a control condition in patients with clinically diagnosed major depression.
Mason et al., (2022) [172]	Treating Young Adult Depression With Text-Delivered CBT: A Pilot Randomized Clinical Trial	Text-delivered CBT is feasible, acceptable, and efficacious for treating depression in young adults.	Total: 102	-The intervention was a 4-week text-message-delivered CBT (CBT-txt) that consisted of 197 text messages delivered at an average frequency of 12 texts every other day.	-The CBT-txt intervention would be feasible, acceptable, and efficacious for treating depression in young adults. -Participants receiving the CBT-txt intervention would be more likely to have minimal or mild depressive symptoms at 2 months compared to a waitlist control group. -The CBT-txt intervention would be more effective at reducing depressive symptoms for participants with more severe depressive symptoms at baseline.
Mohr et al., (2011) [173]	Telephone-administered CBT for veterans served by community-based outpatient clinics	This study found that telephone-administered CBT was not effective in treating depression in veterans served by community-based outpatient clinics.	Total: 85	-The intervention was telephone-administered CBT (T-CBT), which participants received for 16 sessions over 20 weeks.	-T-CBT would be effective for treating depression in veterans served by community-based outpatient clinics (CBOCs) outside of major urban areas. -T-CBT would be more effective than treatment as usual for treating depression in this population.
Mohr et al., (2019) [174]	A randomized non-inferiority trial evaluating remotely-delivered stepped care for depression using Internet CBT and telephone CBT	Stepped care with Internet and telephone CBT is non-inferior, less costly, but less satisfactory to patients than telephone CBT alone for depression.	Total: 270 -Stepped care: 134 -tCBT: 136	-iCBT with telephone and messaging support -Telephone-administered CBT (tCBT) for non-responders to the iCBT	-The stepped-care program was non-inferior to tCBT alone in terms of treatment effectiveness. -The stepped-care program was less costly to deliver than tCBT alone. -The stepped-care program was as acceptable to patients as tCBT alone.
Moreira et al., (2015) [175]	The effect of proinflammatory cytokines in CBT	CBT is more effective than Narrative Cognitive Therapy in reducing proinflammatory cytokines in major depressive disorder.	Total: 97	-The interventions were Narrative Cognitive Therapy (NCT) and CBT.	-CBT and NCT would have different effects on the circulating levels of the proinflammatory cytokines IL-6 and TNF-α in individuals with major depressive disorder (MDD). -CBT would be more effective than NCT in reducing the serum levels of the proinflammatory cytokines IL-6 and TNF-α in individuals with MDD.
Naik et al., (2019) [176]	Effect of Telephone-Delivered Collaborative Goal Setting and Behavioral Activation vs Enhanced Usual Care for Depression Among Adults With Uncontrolled Diabetes	Telephone-delivered collaborative goal setting and behavioral activation are compared to enhanced usual care for depression in adults with uncontrolled diabetes.	Total: 225 -Intervention (HOPE): 135 participants -Control (EUC): 90 participants	-The HOPE intervention was a 6-month telephone-delivered program that included nine sessions focused on collaborative goal setting, behavioral activation, and skill-building to target both depression and diabetes self-care, followed by 6 months of usual care without the HOPE coaches.	-Telephone-delivered collaborative goal setting and behavioral activation (the HOPE intervention) will be more effective than enhanced usual care in improving depression symptoms among US veterans with uncontrolled diabetes. -Telephone-delivered collaborative goal setting and behavioral activation (the HOPE intervention) will be more effective than enhanced usual care in improving glycemic control (HbA1c) among US veterans with uncontrolled diabetes.
Newby et al., (2017) [177]	Web-Based CBT for Depression in People With Diabetes Mellitus: A RCT	Internet-based CBT is an effective treatment for depression in people with diabetes.	Total: 91	-The intervention was a 6-lesson web-based CBT (iCBT) program delivered over 10 weeks, with therapist support provided by phone and e-mail. -The intervention was entirely web-based with no in-person components, and 27 out of 42 participants (66%) completed the full program.	-The Internet-based CBT (iCBT) program would be effective in improving depression, diabetes-related distress, and glycemic control in people with comorbid major depressive disorder and diabetes mellitus. -The iCBT program would also lead to improvements in general distress, disability, anxiety, and somatization.
Nicholas et al., (2019) [178]	Stepping Up: Predictors of ‘Stepping’ within an iCBT Stepped-Care Intervention for Depression	Pre-treatment depression severity and treatment preference predict the likelihood of stepping up to more intensive intervention in iCBT for depression.	Total: 312	-iCBT program called ThinkFeelDo, a modular CBT-based online program with psychoeducation and skill-building exercises, delivered for up to 20 weeks with weekly coaching from a graduate-level therapist. -tCBT (telephone-based CBT) program, a more intensive intervention involving weekly 1-h phone sessions with a therapist and a workbook, which participants were “stepped up” to if they did not improve with the initial iCBT program.	The study did not test any specific hypotheses but rather conducted an exploratory analysis to identify pre-treatment factors associated with “stepping up” to a more intensive intervention (tCBT) within a stepped-care model with iCBT as the initial intervention.
Nicol et al., (2022) [179]	Chatbot-Delivered CBT in Adolescents With Depression and Anxiety During the COVID-19 Pandemic: Feasibility and Acceptability Study	This pilot study demonstrated the feasibility, acceptability, and usability of a chatbot-delivered CBT app for adolescents with depression and anxiety during the COVID-19 pandemic.	Total: 17 -Intervention group: 10 -Waitlist control group: 7	-The intervention in this study is the W-GenZ app, which delivers CBT (CBT), interpersonal psychotherapy for adolescents (IPT-A), and some elements of dialectical behavior therapy (DBT) via brief, self-guided conversations with a conversational agent named Woebot. -The app includes mood tracking, tailored conversations, CBT-based psychoeducation and tools, and daily push notifications to prompt users to check-in. The app uses AI to personalize the program for each user’s needs.	-The app would be acceptable and easy to use for adolescents. -Use of the app would lead to greater improvement in symptoms for adolescents with depression and anxiety compared to a waitlist control group.
O’Mahen et al., (2013) [180]	A Pilot RCT of CBT for Perinatal Depression Adapted for Women with Low Incomes	This pilot study examined the feasibility and symptom outcomes of modified CBT for perinatal depression in low-income women.	Total: 55 -mCBT: 30 -TAU: 25	-The intervention was a modified version of CBT (mCBT) for perinatal depression, consisting of up to 12 individual 50 min sessions. -The intervention included an initial engagement session with Motivational Interviewing, followed by three main treatment modules: Behavioral Activation, Cognitive Restructuring, and Interpersonal Support. -The intervention was adapted for the perinatal period and delivered to a low-income, racially diverse sample.	-CBT modified for the perinatal period (mCBT) will be more effective at reducing depressive symptoms compared to treatment as usual (TAU) in a sample of low-income, racially diverse perinatal women with major depressive disorder. -Demographic and psychological factors will be associated with the feasibility (e.g., adherence, acceptability) of the mCBT intervention. -A greater proportion of women in the mCBT group will experience reliable and clinically significant improvement in depressive symptoms compared to the TAU group.
Pigeon et al., (2023) [181]	A two-phase, prescriptive comparative effectiveness study to optimize the treatment of co-occurring insomnia and depression with digital interventions	This study aims to develop and evaluate an individualized intervention rule for prescribing the optimal digital treatment of co-occurring insomnia and depression.	Total: 2300	-Digital CBT for insomnia (CBT-I) -Digital CBT for depression (CBT-D) -Sequential digital CBT-I followed by digital CBT-D (CBT-I + D) -Sequential digital CBT-D followed by digital CBT-I (CBT-D + I) The duration of the interventions was 12 weeks.	-There are differences in treatment effects between single and sequential digital interventions for co-occurring insomnia and depression. -An individualized intervention rule (IIR) can identify the optimal digital treatment for each individual, compared to randomization to one of the four CBT interventions.
Richards et al., (2020) [182]	A pragmatic randomized waitlist-controlled effectiveness and cost-effectiveness trial of digital interventions for depression and anxiety	Internet-delivered CBT is effective and potentially cost-effective for treating depression and anxiety within stepped-care mental health services.	Total: 361 -iCBT intervention: 241 -Waiting-list control: 120	The intervention was Internet-delivered CBT (iCBT) delivered over an 8-week period.	-iCBT is effective for treating depression and anxiety symptoms when integrated into the IAPT stepped-care model. -iCBT is cost-effective when integrated into the IAPT stepped-care model.
Riese et al., (2021) [183]	Personalized ESM monitoring and feedback to support psychological treatment for depression: a pragmatic RCT (Therap-i)	The study aims to investigate the efficacy of a personalized Experienced Sampling Methodology (ESM) and feedback module integrated into outpatient psychotherapeutic treatment for decreasing depressive symptoms in unresponsive or relapsing patients.	Total: 100 -Intervention arm: 50 -Control arm: 50	-8 weeks of personalized Experienced Sampling Methodology (ESM) monitoring with five assessments per day -3 feedback sessions on the ESM data, discussed between the patient, clinician, and researcher	-The Therap-i module will decrease depressive symptoms in patients with major depressive disorder (MDD) who are unresponsive or relapsing -The Therap-i module will improve general functioning, therapeutic working alliance, and illness perception in patients with MDD who are unresponsive or relapsing.
Ritvo et al., (2021) [184]	Online Mindfulness-Based CBT Intervention for Youth With Major Depressive Disorders: RCT	Online mindfulness-based CBT combined with standard psychiatric care is effective for treating major depressive disorder in youth.	Total: 45	-Online access to 24 CBT-M workbook chapters and 56 mindfulness instruction videos through the NexJ Connected Wellness platform -24 h of navigation coaching delivered via phone and text message over 6 months -A Fitbit-HR Charge 2 device to track physical activity and heart rate, integrated with the NexJ Connected Wellness software	-Online CBT-M with weekly coach interactions and standard psychiatric care is superior to standard psychiatric care alone in improving outcomes for youth with major depressive disorder. -Intervention participants will demonstrate significant improvements in primary outcomes (e.g., depression, anxiety, pain, mindfulness) compared to waitlist controls receiving only standard psychiatric care.
Roepke et al., (2015) [185]	RCT of SuperBetter, a Smartphone-Based/Internet-based Self-Help Tool to Reduce Depressive Symptoms	A smartphone-based/Internet-based self-help tool called SuperBetter was effective in reducing depressive symptoms.	Total: 283 -CBT-PPT SB: ~94 -General SB: ~94 -Waiting list control: ~94	-CBT-PPT SB: A version of the SuperBetter app using CBT and positive psychotherapy strategies to target depression. -General SB: A general version of the SuperBetter app focused on self-esteem and acceptance. Participants in the two SB intervention groups were instructed to use the app for 10 min per day for 1 month.	-SB participants would achieve greater reductions in depressive symptoms (CES-D scores) compared to the waitlist control group, both at post-test and follow-up. -The CBT-PPT version of SB would be more effective than the General SB version in reducing depressive symptoms.
Rosso et al., (2017) [186]	Internet-based CBT for major depressive disorder: A RCT	The Sadness Program, a technician-assisted Internet-based CBT intervention, is effective for treating major depressive disorder.	Total: 77 -iCBT: 37 -MAC: 40	-The intervention was a 10-week Internet-based CBT (iCBT) program called the “Sadness Program” that participants accessed 6 times over the 10 weeks. -The program consisted of 6 sequential CBT lessons presented in an illustrated cartoon format, with participants completing self-report measures before each lesson. -Participants could download lesson summaries, homework, and supplemental resources. -The program was self-paced but had to be completed within 10 weeks, with at least 5 days between lessons.	-Participants assigned to the Internet-based CBT (iCBT) group would show a greater reduction in depression symptoms and higher rates of clinical response and remission compared to the monitored attention control (MAC) group, using both interviewer-rated and self-report measures. -The effects of iCBT would be moderated by baseline depression severity, number of prior depressive episodes, and anxiety disorder comorbidity.
Sauer-Zavala et al., (2022) [187]	A SMART approach to personalized care: preliminary data on how to select and sequence skills in transdiagnostic CBT	Personalized skill selection and sequencing in transdiagnostic CBT may be feasible but did not substantially impact symptom trajectories.	Total: 70	-The intervention was the Unified Protocol for Transdiagnostic Treatment of Emotional Disorders (UP), which consisted of five core skills (Understanding Emotions, Mindful Emotion Awareness, Cognitive Flexibility, Countering Emotional Behaviors, and Confronting Physical Sensations) delivered in individual, weekly 45–60 min sessions. -Participants were randomly assigned to receive the modules in either a personalized sequence based on their relative strengths and deficits or in the standard order prescribed in the UP manual. -Participants were also randomly assigned to receive either a full course of 12 sessions or a brief course of 6 sessions.	-Patients in the Capitalization condition would show steeper trajectories of symptom improvement compared to the Compensation and Standard conditions. -A personalized selection of UP modules delivered across six sessions would lead to similar outcomes as the full 12-session course of the standard UP module sequence.
Schramm et al., (2024) [188]	Algorithm-based modular psychotherapy vs. CBT for patients with depression, psychiatric comorbidities, and early trauma: a proof-of-concept RCT	Algorithm-based modular psychotherapy complementing CBT shows promise for treating depression with comorbidities and early trauma.	Total: 70	-Standard CBT alone, consisting of 20 sessions over 16 weeks. -CBT plus additional “transdiagnostic modules” according to a “mechanism-based treatment algorithm” (MoBa), also consisting of 20 sessions over 16 weeks.	-To assess the feasibility of an algorithm-based modular psychotherapy (MoBa) approach -To compare MoBa vs. standard CBT in terms of: -Participants’ and therapists’ overall satisfaction and ratings of therapeutic alliance –Efficacy –Impact on early trauma-related transdiagnostic mechanisms –Safety.
Scogin et al., (2018) [189]	Effects of Integrated Telehealth-Delivered CBT for Depression and Insomnia in Rural Older Adults	Integrated CBT for depression and insomnia delivered via videoconference improved sleep but had equivocal effects on depression in rural older adults.	Total: 40	-10 sessions of integrated CBT for depression and insomnia (CBT-D + CBT-I) were delivered via videoconference, with roughly 25 min devoted to each component per session.	-Depressive symptoms (as measured by the HAM-D) would show significantly greater improvements from baseline to post-treatment in the integrated CBT-D + CBT-I group compared to the usual care control group. -Sleep quality (as measured by the ISI) would show significantly greater improvements from baseline to post-treatment in the integrated CBT-D + CBT-I group compared to the usual care control group.
Stubbings et al., (2013) [190]	Comparing In-Person to Videoconference-Based CBT for Mood and Anxiety Disorders: RCT	CBT delivered via videoconference is as effective as in-person therapy for treating mood and anxiety disorders.	Total: 26 -Videoconferencing condition: 14 participants (six males, eight females) -In-person condition: 12 participants (five males, seven females)	-The intervention was 12 weekly 1-h sessions of individualized CBT, with an additional follow-up session 6 weeks after the 12th session. The CBT interventions were tailored to the specific disorder of each participant, using manualized protocols as a guide, and included psychoeducation, symptom monitoring, cognitive restructuring, and exposure exercises.	-There would be no significant differences in clinical outcomes (symptoms of depression, anxiety, stress, and quality of life) between the in-person and videoconference-based CBT conditions. -The videoconference-based CBT condition would produce comparable changes in quality of life as the in-person condition. -There would be no significant differences in the working alliance between the in-person and videoconference-based CBT conditions.
Swartz et al., (2023) [191]	Randomized trial of brief interpersonal psychotherapy and CBT for depression delivered both in-person and by telehealth	Brief interpersonal psychotherapy and CBT for depression are effective when delivered in-person or via telehealth, with telehealth potentially improving therapy adherence.	Total: 77	-8 sessions of CBT, delivered either in-person (CBT-IP) or via telehealth (CBT-TH) -8 sessions of Interpersonal Psychotherapy (IPT), delivered either in-person (IPT-IP) or via telehealth (IPT-TH)	-Whether there are differential outcomes in terms of changes in depression symptoms (HRSD-17) between the CBT and IPT groups and between the in-person (IP) and telehealth (TH) delivery phases. -Whether the working alliance (WAI) differs between the CBT and IPT groups and between the IP and TH delivery phases. -Whether therapy adherence, as measured by the number of completed therapy sessions, differs between the IP and TH delivery phases.
Szigethy et al., (2023) [192]	Efficacy of a digital mental health intervention embedded in routine care compared with treatment as usual in adolescents and young adults with moderate depressive symptoms: protocol for randomised controlled trial	This protocol describes an RCT evaluating a digital cognitive-behavioral intervention for depressed adolescents and young adults compared to usual care.	Total: 750 -dCBI + TAU: 450 participants (150 per site) -TAU alone: 300 participants (100 per site)	-The intervention is a digital cognitive- behavioral intervention (dCBI) called RxWell, which is provided in addition to treatment as usual (TAU). RxWell is a mobile app that provides users with brief (5–10 min) skill-building techniques such as relaxation, behavioral activation and exposure, distress tolerance, cognitive reframing, and mindfulness meditation for anxiety and depression. -RxWell also includes an integrated digital health coach who communicates with users via asynchronous, secure messaging within the app to reinforce CBT principles, guide goal setting, motivate users, and provide support.	-The digital CBT intervention will reduce depressive symptoms as measured by both self-report (PHQ-9) and clinician-rated (CDRS-R) scales. -The digital CBT intervention will lead to reductions in suicidal ideation anxiety, and improvements in quality of life and functioning. -Certain baseline characteristics, such as race, socioeconomic class, geography, and participant expectancy, may moderate the treatment response or predict adherence to the digital CBT intervention.
Thase et al., (2020) [193]	Improving Cost-effectiveness and Access to CBT for Depression: Providing Remote-Ready, Computer-Assisted Psychotherapy in Times of Crisis and Beyond	Computer-assisted CBT is a cost-effective approach that can improve access to treatment for depression.	Total: 154 -Standard CBT: 77 -CCBT: 77	-Conventional CBT: 20 sessions of 50 min each, for a total of 16 h and 40 min of therapist time over 16 weeks. -CCBT: A 50 min introductory session, 11 25 min sessions (for a total of 5.5 h of therapist contact), and nine Internet-delivered modules that took 30–60 min each for participants to complete.	-The therapist-supported CCBT method using the Good Days Ahead program would be more cost-effective compared to standard CBT. -The CCBT method using the Good Days Ahead program would have comparable clinical efficacy to standard CBT, despite requiring less therapist contact time.
Titov et al., (2015) [194]	Disorder-specific versus transdiagnostic and clinician-guided versus self-guided treatment for major depressive disorder and comorbid anxiety disorders: A RCT	Disorder-specific and transdiagnostic CBT, delivered in clinician-guided or self-guided formats, are all effective at treating major depressive disorder and comorbid anxiety disorders.	Total: 290	-Transdiagnostic cognitive-behavioral therapy (TD-CBT), delivered in an Internet-based format -Disorder-specific cognitive-behavioral therapy (DS-CBT), delivered in an Internet-based format -Clinician-guided cognitive-behavioral therapy (CG-CBT), delivered in an Internet-based format -Self-guided cognitive-behavioral therapy (SG-CBT), delivered in an Internet-based format.	-Comparing the effectiveness of disorder-specific CBT (DS-CBT) and transdiagnostic CBT (TD-CBT) in treating major depressive disorder (MDD) and comorbid anxiety disorders. -Comparing the effectiveness of clinician-guided CBT (CG-CBT) and self-guided CBT (SG-CBT) in treating MDD and comorbid anxiety disorders.
Twomey et al., (2020) [195]	Effectiveness of a tailored, integrative Internet intervention (deprexis) for depression: Updated meta-analysis	The tailored, integrative digital intervention deprexis is effective for reducing depressive symptoms over 8–12 weeks.	Total: 2901	-The intervention was the deprexis program, a tailored, integrative Internet-based intervention for depression that was used adjunctively to other forms of depression treatment. -The deprexis program included interactive modules on topics like positive psychology and interpersonal skills, and it engaged users in simulated dialogues where they could select responses that would then tailor the subsequent content. -The duration of the deprexis intervention was 8–12 weeks.	-The effectiveness of deprexis when used as an adjunct to inpatient or outpatient depression treatment -The effectiveness of deprexis compared to an active control condition (rather than just waitlist or treatment-as-usual) -Whether the effectiveness of deprexis was moderated by the setting (clinical vs. community), developer involvement, or provision of personal support.
Venkatesan et al., (2022) [196]	Improvements in Depression Outcomes Following a Digital CBT Intervention in a Polychronic Population: Retrospective Study	A digital CBT intervention improved depression outcomes in a population with chronic physical conditions.	Total: 1512	-Structured digital lessons, activities, and practices based on CBT principles -An initial consultation with a licensed therapist for assessment and treatment planning -Weekly 12-week video/audio consultations with the therapist, followed by monthly sessions for up to 1 year -Completion of homework and use of a thought tracker in the app between sessions.	-Participants who completed the Vida CBT Program would show a reduction in depressive symptoms -Measures of program usage would be positively associated with improvements in depressive symptoms -Additionally, the study conducted an exploratory analysis to evaluate whether improvements in depression would be positively associated with greater weight loss among participants concurrently enrolled in a Vida physical health program.
Vernmark et al., (2010) [197]	Internet administered guided self-help versus individualized e-mail therapy: A randomized trial of two versions of CBT for major depression	Guided self-help and individualized e-mail therapy are both effective forms of Internet-delivered CBT for major depression.	Total: 88	-Guided self-help CBT delivered via the Internet, with weekly modules, homework assignments, and brief therapist support. -Individualized e-mail therapy, where all emails were written specifically for each patient. -Both interventions lasted for 8 weeks.	-Guided self-help Internet-delivered CBT would be effective for treating major depression -Individualized e-mail therapy Internet-delivered CBT would be effective for treating major depression
Watanabe et al., (2015) [198]	Adding smartphone-based CBT to pharmacotherapy for major depression (FLATT project): study protocol for an RCT	This study protocol evaluates the effectiveness of adding a smartphone-based CBT program to antidepressant treatment for treatment-resistant depression.	Total: 164	-The intervention is a 9-week smartphone-based CBT program called the “Kokoro-App” that is provided in addition to switching antidepressant medication.	-The effectiveness of switching antidepressants and starting a smartphone-based CBT program, compared to switching antidepressants only, in patients with treatment-resistant depression.
Weisz et al., (2009) [199]	CBT versus usual clinical care for youth depression: an initial test of transportability to community clinics and clinicians	CBT for youth depression showed advantages over usual clinical care in parent engagement, reduced use of medication/services, and cost, but no difference in depression outcomes.	Total: 57	-Usual Care (UC): Details on frequency, duration, and dose not provided -CBT using the PASCET program: 1-day, 6-h training, 30 min of weekly supervision, 10–15 individual sessions	-CBT would be superior to usual care (UC) on clinical outcomes (depression symptoms and diagnoses) -CBT would be superior to UC on therapeutic alliance (consumer response) -CBT would have lower cost and require less use of additional clinical services compared to UC.
Wiles et al., (2014) [200]	Clinical effectiveness and cost-effectiveness of CBT as an adjunct to pharmacotherapy for treatment-resistant depression in primary care: the CoBalT randomised controlled trial	CBT, as an adjunct to usual care, including pharmacotherapy, is effective and cost-effective for treatment-resistant depression in primary care.	Total: 469 -234 in the intervention group and -235 in the usual care group.	-The intervention was 12–18 sessions of CBT in addition to usual care, which included pharmacotherapy.	-CBT as an adjunct to usual care (including pharmacotherapy) is clinically effective in reducing depressive symptoms in patients with treatment-resistant depression, compared to usual care alone. -CBT as an adjunct to usual care is cost-effective compared to usual care alone.
Wilhelm et al., (2024) [201]	Feasibility, Acceptability, and Preliminary Efficacy of a Smartphone App–Led CBT for Depression Under Therapist Supervision: Open Trial	A smartphone-led CBT for depression, supported by brief teletherapy, is feasible, acceptable, and shows preliminary efficacy.	Total: 28	-An 8-week smartphone-based CBT program called “Mindset for Depression” -8 brief (16–25 min) videoconferencing sessions with a therapist, conducted weekly -Ability to communicate with the therapist between sessions through secure in-app messaging	-The treatment (Mindset for Depression app with brief therapist support) would be feasible and acceptable. -The treatment would yield statistically significant reductions in depression symptom severity (primary outcome) and improvements in functioning and quality of life (secondary outcomes) from baseline to post-treatment.
Wilkinson et al., (2021) [202]	CBT to Sustain the Antidepressant Effects of Ketamine in Treatment-Resistant Depression: A Randomized Clinical Trial	CBT may help sustain the antidepressant effects of ketamine in treatment-resistant depression.	Total: 42	-The intervention was six intravenous infusions of ketamine over 3 weeks. Some participants then received 14 weeks of CBT following the ketamine infusions.	-The feasibility and efficacy of CBT (CBT) following intravenous ketamine treatment in sustaining the antidepressant effects in patients with treatment-resistant depression. -The CBT group will show greater sustained improvement in depressive symptoms compared to the treatment-as-usual (TAU) group, as measured by the Quick Inventory of Depressive Symptomatology. -Ketamine responders will show improvements in emotional cognitive function, as measured by the emotional N-back task, whereas non-responders will not.
Williams et al., (2013) [203]	Combining Imagination and Reason in the Treatment of Depression	Combining computerized cognitive-bias modification and Internet-based CBT is an effective treatment for depression.	Total: 69 -Intervention group: 38 -Wait-list control group: 31	-CBM-I (cognitive-bias modification targeting interpretation): 7 sessions, each 20 min long, completed daily over 1 week. -iCBT (Internet-based cognitive-behavioral therapy): 6 online lessons representing CBT, as well as homework assignments and supplementary resources, delivered over 10 weeks.	-The CBM-I intervention alone would lead to significant reductions in depressive symptoms and distress compared to a waitlist control group. -The change in interpretation bias (as measured by the AST-D and SST) would mediate the reduction in depressive symptoms following the CBM-I intervention. -The combined CBM-I and iCBT intervention would lead to greater improvements in depressive symptoms, distress, disability, anxiety, and repetitive negative thinking compared to the waitlist control group.
Wright et al., (2022) [204]	Effect of Computer-Assisted CBT vs Usual Care on Depression Among Adults in Primary Care	Computer-assisted CBT was more effective than usual care in treating depression in primary care patients.	Total: 240	-The intervention was computer-assisted CBT using the 9-lesson “Good Days Ahead” (GDA) program, provided for 12 weeks along with up to 12 telephonic support sessions with a master’s level clinician, averaging 20 min per week. -The CCBT intervention was provided in addition to the participants’ usual care (TAU). -The study also provided low-cost laptops with Internet access to 17 out of 175 participants (9.7%) who lacked Internet access.	-CCBT plus TAU would be more effective at improving depression outcomes compared to TAU alone. -CCBT would be feasible and effective in a primary care population with low education, reading skills, and/or Internet access. -Certain factors (e.g., baseline symptoms, reading level, education) would be associated with differential treatment outcomes between CCBT and TAU. -CCBT would have lower medical care utilization costs compared to TAU.
Xiang et al., (2023) [205]	Layperson-Supported Internet-Delivered CBT for Depression Among Older Adults	Layperson-supported Internet-delivered CBT is a promising intervention for treating depression in older adults.	Total: 103	-The intervention in this study was Empower@Home, a digital CBT (CBT) intervention for geriatric depression. Participants with depressive symptoms (PHQ-9 ≥ 5) underwent a 9-session remote intervention supported by a lay coach. On average, participants completed 8.5 of the nine sessions.	-The Empower@Home intervention would be feasible and acceptable for older adults with depression. -The Empower@Home intervention would lead to a reduction in depressive symptoms, with a larger effect for those with moderate depression at baseline. -The Empower@Home intervention would lead to improvements in other mental health and well-being outcomes, such as anxiety, social isolation, loneliness, and behavioral activation.
Young et al., (2020) [206]	Personalized Depression Prevention: A RCT to Optimize Effects Through Risk-Informed Personalization	Personalizing depression prevention by matching interventions to youths’ psychosocial vulnerabilities enhances effects compared to a one-size-fits-all approach.	Total: 204	-Coping With Stress (CWS), a cognitive-behavioral program -Interpersonal Psychotherapy-Adolescent Skills Training (IPT-AST), an interpersonal program	-Matched adolescents (those receiving an intervention that addressed their specific psychosocial vulnerabilities) would show greater decreases in depressive symptoms compared to mismatched adolescents. -There would be no significant difference in rates of depressive disorders between matched and mismatched adolescents.
Zaferanieh et al., (2023) [207]	Web-Based MBCT for Adults With a History of Depression: Protocol for an RCT	This study protocol aims to examine the feasibility and efficacy of web-based mindfulness-based cognitive therapy for the treatment of depression.	Total: 128	-The intervention is an 8-week web-based MBCT program, in addition to treatment as usual.	-Web-based MBCT is feasible for the treatment of depression -Web-based MBCT is efficacious in reducing depression symptoms and psychiatric distress -Web-based MBCT will improve perceived stress and mindfulness -Measures of feasibility (adherence, retention, attendance, engagement) will indicate that web-based MBCT is feasible -Mindfulness, self-practice, and executive functioning skills will mediate the intervention outcomes.

## Abbreviations

CBT	Cognitive-Behavioral Therapy
NG-CBT	Next-Generation Cognitive-Behavioral Therapy
RCT	Randomized Controlled Trial
AI	Artificial Intelligence
VR	Virtual Reality
TAU	Treatment As Usual
ICBT	Internet Cognitive-Behavioral Therapy
DCT	Digital Cognitive Therapy
MBCT	Mindfulness-Based Cognitive Therapy
EBT	Evidence-Based Therapy
PST	Problem-Solving Therapy
PHQ-9	Patient Health Questionnaire-9
dCBT-I	Digital Cognitive-Behavioral Therapy for Insomnia
EsCIT	Escitalopram
T-CBT	Telephone Cognitive-Behavioral Therapy
CCBT	Conventional Cognitive-Behavioral Therapy
RCBT	Religiously-Integrated Cognitive-Behavioral Therapy
PAI	Personalized Advantage Index
PRISMA	Preferred Reporting Items for Systematic Reviews and Meta-Analyses
CBT-I	Cognitive-Behavioral Therapy for Insomnia
CCBT	Computerized Cognitive-Behavioral Therapy
CCBT	Computerized Cognitive-Behavioral Therapy
TAU	Treatment As Usual
ICBT-A	Internet Cognitive-Behavioral Therapy for Antenatal Depression
CBT-txt	Cognitive-Behavioral Therapy Delivered via Text
BBI	Biobehavioral Intervention
